# Self-Sensing Cementitious Composites: Review and Perspective

**DOI:** 10.3390/nano11092355

**Published:** 2021-09-10

**Authors:** Zere Bekzhanova, Shazim Ali Memon, Jong Ryeol Kim

**Affiliations:** Department of Civil and Environmental Engineering, School of Engineering and Digital Sciences, Nazarbayev University, Nur-Sultan 010000, Kazakhstan; zere.bekzhanova@alumni.nu.edu.kz (Z.B.); jong.kim@nu.edu.kz (J.R.K.)

**Keywords:** self-sensing, cementitious composites, electrical resistivity, damage detection, structural health monitoring

## Abstract

Self-sensing concrete (SSC) has been vastly studied for its possibility to provide a cost-effective solution for structural health monitoring of concrete structures, rendering it very attractive in real-life applications. In this review paper, comprehensive information about the components of self-sensing concrete, dispersion methods and mix design, as well as the recent progress in the field of self-sensing concrete, has been provided. The information and recent research findings about self-sensing materials for smart composites, their properties, measurement of self-sensing signal and the behavior of self-sensing concrete under different loading conditions are included. Factors influencing the electrical resistance of self-sensitive concrete such as dry-wet cycle, ice formation and freeze thaw cycle and current frequency, etc., which were not covered by previous review papers on self-sensing concrete, are discussed in detail. Finally, major emphasis is placed on the application of self-sensing technology in existing and new structures.

## 1. Introduction

Concrete is the second most widely used material, with around 30 million tons consumed annually. It finds its application in pavements, buildings, nuclear power plants and dams, [1] etc. Different types of concrete-like high strength, lightweight, recyclable and ultrahigh performance concrete have been rapidly developed and implemented in the construction industry [2]. However, the cracking, deterioration, deformation and scaling in concrete are inevitable consequences of long-term environmental and mechanical impacts that further reduce the concrete lifetime. Hence, in recent decades, the need to monitor and evaluate concrete performance during its maintenance or service life with the help of condition assessing tools and various materials has attracted more attention. The structural health monitoring (SHM) technology provides the ability to monitor the condition to ensure the safety of the infrastructure during its service life. Electric-resistance strain gauges, piezoelectric strain sensors and optic sensors are mainly implemented in construction as sensors for evaluation and monitoring purposes; however, their usage has some drawbacks such as poor durability and low sensitivity [3]. Recent developments of smart materials including cement-based sensors could resolve these issues.

The introduction of self-sensing concrete could help to sense and monitor concrete structures, thus allowing the improvement of safety, durability, serviceability and reliability. Intrinsic self-sensing concrete (ISSC) consists of incorporating functional fillers such as carbon black (CB), carbon fibers (CF), graphite powder (GP), carbon nanomaterials and others into conventional concrete, thus improving mechanical properties and providing the ability to monitor damage and stress/strain [4]. Different research studies were conducted in order to examine the performance of SSC, including its implementation for traffic monitoring [5], corrosion monitoring [6], strain sensing [7], seismic damage monitoring [8] and so on. In addition, many research studies were conducted to determine the properties of SSC incorporated with different functional fillers such as carbon fiber [9], carbon nanofiber [10], carbon nanotube [11,12,13], graphene nanoplatelets [14], steel fibers [15], glass fiber [16] and so on. This shows that over the past two decades, many research papers have been concentrated on the development and application of SSC.

Overall, the properties of SSC are influenced by many factors and initial conditions such as material selection, particularly the matrix material, functional filler, dispersion material and further dispersion processes. An important step in the preparation procedure is the mix design, which includes the selection of the optimal concentration of functional fillers that differs according to the material. Hence, the final SSC design is different and highly dependent on the properties of the fillers. Similarly, the methods and materials used to monitor the sensing abilities of concrete differ and are mainly selected based on the preferences of the researchers. In studies related to the application of SSC, the final results vary according to the loading conditions and depend on the factors affecting its electrical resistivity. In addition, the conductive mechanism in concrete changes depending on the type of load, which affects the concentration of the functional filler in certain areas. By improving the properties of SSC, the real-life application of it has a promising potential in various fields, which can be demonstrated by practical experiments carried out by researchers.

In this review paper, a comprehensive approach to the use of self-sensitive concrete starting from material selection to its application is described using existing literature and research. It is novel in the sense that it includes an up-to-date detailed description of the experimental investigations performed in the area of SSC. The details of the factors influencing its sensing properties such as dry-wet cycle, ice formation and freeze thaw cycle and current frequency, etc., which were not covered by previous review papers on SSC, are incorporated. Finally, this review paper aims at providing readers extensive up-to-date information about the potential application of self-sensing technology. The review paper starts with providing information about the components of SSC, dispersion methods and mix design. The details about the measurement of self-sensing signal and the conductive mechanism of SSC with and without external loading are provided in Section 3 and Section 4. Section 5 presents information about the behavior of SSC under different loading conditions and the factors influencing the electrical resistivity including functional filler concentration, the geometrical shape of functional fillers, temperature, loading rate and moisture, etc. Comprehensive information and research findings about self-sensing materials including carbon fibers and carbon nanomaterials for smart composites and their properties have been included in Section 6. Finally, the information regarding the potential application of this technology in existing and new structures, together with recommendations for future research, is covered in Section 7 and Section 8.

## 2. Components of Self-Sensing Concrete, Dispersion Methods and Mix Design

Self-sensing function of the concrete is obtained from the piezoresistive effect of functional fillers, which are dispersed in the matrix phase and form a conductive network. Therefore, when concrete is subjected to some force, the network alters, and it results in a change in electrical resistance. Overall, the self-sensing concrete has a very complex structure, and for a detailed description of the basic concepts, the next section focuses on matrix material, functional filler, dispersion methods, mix preparation and design.

### 2.1. Matrix Material

The composition of SSC includes matrix material, functional filler and dispersion material. Here, the matrix material is usually represented as conventional concrete, and it could be made of cement, mortar and paste [17]. The main function of the concrete matrix is to hold together the functional filler. One of the most commonly used components of the matrix is the Portland cement, which is frequently used for SSC preparation [4]. The sensing ability of the concrete is poor or even sometimes absent; however, this ability directly depends on the mechanical behavior (stress and strain, etc.) and on electrical conduction. The proportion and the mix designs of the matrix material affect the distribution and dispersion of the fillers, thus influencing the total sensing ability of the concrete [4].

### 2.2. Functional Filler

Self-sensing concrete mainly achieves its properties because of the inclusion of conductive functional fillers such as carbon black (CB), short and continuous carbon fibers (CF), steel slag (SS), nickel powder (NP), magnetic fly ash (MFA), graphite powder (GP) and carbon nanomaterials functional fillers such as carbon nanotubes (CNT) and carbon nanofiber (CNF) [17]. Some hybrid fillers can also provide different sensing properties to concrete, which could not be previously achieved by other fillers. All these conducting components provide a path for the current to travel along with the cement matrix, thus forming an electrical network [18]. When the cement composite is subjected to some load, the resistivity in elastic regime, plastic deformation regime and after failure is increased with the increase in the deformation and damage [19]. However, the conducting network and change in the resistivity highly depend on the amount of fillers, distribution and parameters such as morphology (size, shape, surface state, agglomeration and degree of aggregation) and material components [4]. These factors should be considered for the modification of the sensing properties of the concrete. Table 1 shows different fillers’ classifications by their material component, filler shape and scale, conductivity capability, application stile and surface state.

### 2.3. Dispersion Methods

Electrical conductivity plays an essential part in SSC characteristics. According to Heilig [20], this parameter directly depends on the dispersion of functional fillers in the matrix material because the uniform distribution of conductive particles improves the composite’s homogeneity, thus positively affecting the electrical network of the concrete matrix. The challenge is that fibers usually form agglomerates and bundles because of the van der Waals force between fillers [4]. Therefore, effective dispersion methods are required to ensure uniform distribution. 

Different methods could be used for the proper dispersion of fibers and can be divided into physical and chemical methods. The physical methods include processes such as sonication, ball milling and mechanical stirring [21]. Alessandro et al. [22] investigated the influence of various physical methods, including the mechanical mixing and sonication process, to find the most effective method for dispersing fibers in the matrix material. The findings showed that the implementation of the sonication method resulted in better signal quality in electrical resistance measurements, and a desirable level of dispersion was accomplished. 

Chemical methods for dispersion include surfactants and mineral admixtures [21]. According to Han et al. [4], the dispersion of surfactant can be obtained by wetting, electrostatic repulsion and steric hindrance effects, while the mineral admixture dispersion is attained by gradation, adsorption and separation effects. Mineral admixtures and surfactants are both used as dispersion materials; however, for different fibers, different surfactants and admixtures should be considered for better results. Table 2 provides a list of dispersion materials that are usually used for functional filler distribution. There is also a relatively new method where spraying is applied in chemical dispersion [23]. This method would be described further in the carbon nanomaterials section. 

Nevertheless, two methods, including chemical and physical, could be applied at the same time. In the experimental study conducted by Faghih [18] to disperse CNF, the chemical dispersion method, together with ultrasonication, was used to achieve optimal dispersion of fillers. Han et al. [24] evaluated the effect of superplasticizer as a dispersant instead of using sodium dodecyl sulfate (SDS), sodium dodecylbenzene sulfonate (SDBS) and methylcellulose (MC), which have a negative impact on the hydration of cement and further influenced the mechanical properties of cementitious composites by making them weaker. During the experiment, the superplasticizer was firstly mixed with water using a magnetism stirrer, and then fillers (CNT/CNF) were added into a solution where an ultrasonicator was applied. In order to achieve better dispersion, the mixing was performed for 2 h. The study showed that the dispersion of CNT/CNF with superplasticizer effectively distributes fillers uniformly in a cement matrix as it showed strong piezoresistive responses.

### 2.4. Mix Preparation and Design

The mix preparation design for self-sensing concrete follows a similar protocol as in the case of conventional concrete design except for the addition of filler in self-sensing concrete. Due to high surface area of the filler, the workability of the concrete mix could be affected, and the chemicals that are used as dispersant materials could influence the mechanical properties of SSC at the same time. 

The addition of a specific percentage of filler in the mix should be carefully selected to achieve the desired results. For instance, it was found that the increase in CNF from 0.25% to 1.0% volume fraction of the concrete resulted in the decrease in electrical resistance by nearly half of the initial value [18]. Compared to 0.25%, 0.5% and 0.75% addition of CNF in the mixture, the 1.0% volume fraction showed a 39% higher compressive strength than the control mix. It was concluded that the addition of fillers in the correct proportion can help to increase the strength of the concrete mix [25]. Table 3 demonstrates some of the optimal concentration values for different functional fillers. Similarly, the hybrid mixtures could be added in the composite instead of only one type of filler. Findings from the research study of Azhari and Banthia [26] showed that the addition of 15% CF in the paste mixture resulted in lower electrical conductivity compared to the hybrid mixture that contained 15% of CF and 1% of MWCNTs. The hybrid mixture showed a 10% improvement in conductivity compared to the mixture with CF filler alone. Similarly, in another investigation regarding the mixing of fillers with water, the uniform distribution of fibers in concrete was achieved by mixing the fillers with the entire amounts of water instead of only a portion of the water [18]. 

**Figure 1:**
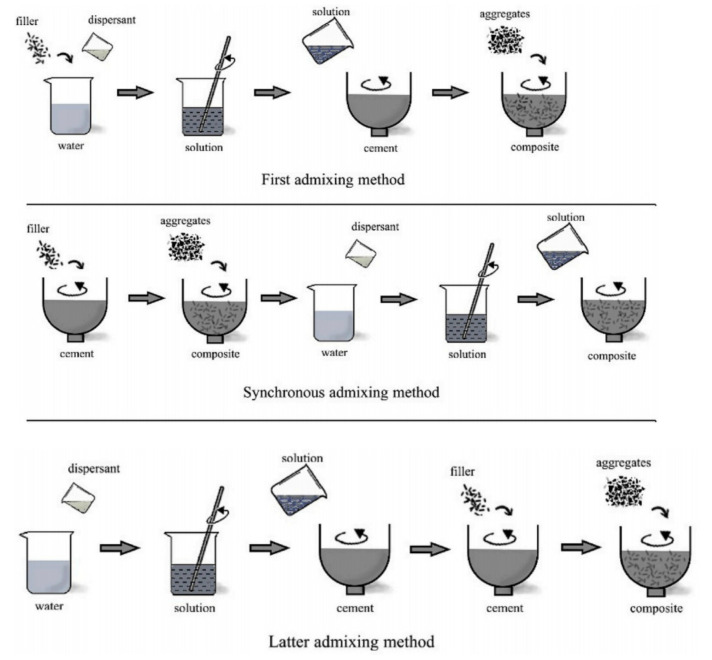
Three mixing procedures. Reproduced with permission from [34], Elsevier, 2019.

In general, there are three types of dispersion processes regarding the addition of functional fillers, and they are classified as the first admixing method, synchronous admixing method and latter admixing method (Figure 1). Mostly, the first admixing mixing method is applicable for fibrous particles where functional fillers are pre-mixed into an aqueous solution, while the latter mixing method is mainly advantageous for larger particle sized fillers where the need for a pre-aqueous solution is not required. In synchronous admixing, which is mostly applicable to a hybrid filler system, cement is firstly mixed together with the first filler, which is then followed by the addition of second filler to this solution [34]. Overall, it can be deduced that the mixing design and the procedure of concrete preparation should be elaborated in advance for the achievement of desired results in the properties of the mix. 

## 3. Measurement of Sensing Signal of Concrete 

**Figure 2:**
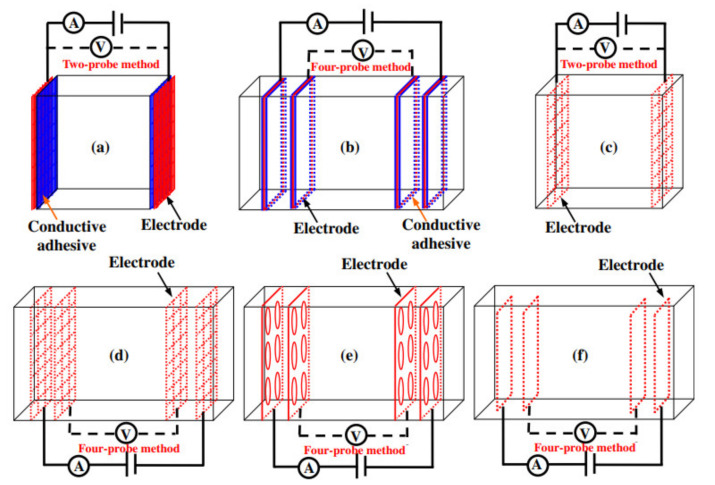
Fixing the style and layout of electrodes in self-sensing concrete; (**a**,**b**): electrodes attached on the surface; (**c**–**f**): embedded mesh, perforated plate or loop electrode. Reproduced with permission from [4], Elsevier, 2015.

There are many factors, such as reactance, electrical resistance, dielectric constant, impedance and capacitance, for determining the sensitivity of the concrete [4]. However, one of the most commonly used methods is electrical resistance/resistivity that is implemented as an indicator of sensitivity. The fibrous conductivity inside cement-based materials affects the electrical resistivity, which changes during deformation, temperature or damage [35]. Therefore, electrodes made of appropriate materials are used to measure electrical conductivity in various configurations. There are three factors (material, fixing location and layout), which should be considered before choosing an electrode. The main characteristics of the material should be its low electrical resistance as well as the stabilized conductive property [4]. These electrodes could be attached to the surface of concrete, embedded inside of the concrete or located in a clipping style. Among them, embedment and attachment are some of the commonly used methods. The measurements can be taken in a two-probe or a four-probe configuration, where both electrodes can be used as current pole and voltage pole in a two-probe method, as shown in Figure 2. In contrast, in four-probe configuration, voltage is detected by the inner probes while the outer probes are used as an electric current detector [16]. The two-probe method is more convenient and simpler to use compared to the four-probe method. However, the four-probe method is mostly preferred due to the accuracy of the results since it is used to measure low resistance values. In addition, the value of the probe’s resistance is not added to the resistance of the tested specimen [36]. In the case of two-probe method, the same two probes are used to supply the voltage and also to record the current flowing from the applied voltage. Therefore, the internal resistance of the multimeter results in inaccuracy as it is added to the actual displayed value.

**Figure 3:**
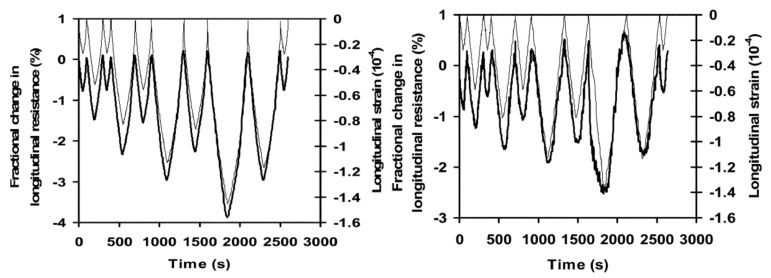
The fractional change in longitudinal resistance (thicker line) and longitudinal strain (thinner line) versus time for four-probe with embedded stainless-steel foils and four-probe connected on top [37].

The research conducted by Wen and Chung [37] compared two types of electrode contact configurations in a 0.5% carbon fiber reinforced cement paste. The first one was a four-probe method with four embedded stainless-steel foils and the second one was the four-probe method with four electrical contacts in the shape of silver paint in combination with copper wire attached on top of the surface. These two methods were tested for the longitudinal resistance during repeated loads on the samples. According to the test results, the values for the signal-to-noise ratio in embedded electrodes was higher compared to four-probe that were placed on top of the surface, as shown in Figure 3. The reason for that could be the mechanical stability of the electrodes that were embedded compared to electrodes that were attached to the top surface during the loading process. The authors found that for strain sensing, in the four-probe method, the embedment of electrodes was better than the configuration with a contact of an electrode on top of the surface. Thus, the greater signal-to-noise level and the results regarding uniformity of the current density showed that the four-probe method with embedded electrodes is superior to the four electrodes on top of the surface.

The authors Wen and Chung [37] performed the same experiment as mentioned above by using the two-probe method. It was found that the two-probe method demonstrated higher electrical resistance compared to the four-probe method. Additionally, the gauge factor of the four-probe method showed higher values, and its variation tends to be less with strain amplitude compared to the two-probe method, thus suggesting more accurate strain sensing capability. Therefore, various researchers such as [3,4,7,18,19,21,38,39,40,41] prefer to use the four-probe method over two-probe in their studies. 

The influence of DC and AC electrical circuits on self-sensing concrete containing conductive particles should also be mentioned. The use of DC has been shown to be economically viable and useful for laboratory applications to characterize cement composites [2]. It should be noted, however, that when the DC is implemented in the sensory system, the sensors with conductive particles electrically charge themselves. This can be explained by their capacitive performance, rather than inductive performance [21]. Consequently, the electrical polarization in self-sensing structural materials, which occurs due to the charging ability, makes it difficult to accurately measure the changes in electrical resistance using direct current because it creates an inherent time-based drift. The drift is expressed in terms of the increase in resistance since the start of the resistance measurement [36]. One method to reduce or negate this effect is to let resistance plateau after full polarization by applying a DC voltage potential before loading the sensing material [4]. Another method to avoid electrical polarization is to use AC signals. However, in AC signals, there are capacitive and skin effects that affect the fluctuations in the measured resistance. By using a higher AC frequency, the capacitive effect can be decreased, while the effect from skin comes into effect when the frequency achieves a threshold [2]. Therefore, proper choice of frequency is also one of the important factors to consider.

## 4. Conductive Mechanism of Self-Sensing Concrete 

Functional fillers inside of SSC behave differently during loading and non-loading conditions. Different concentrations of filler materials in SSC could be divided into various zones that have different electrical resistivity.

### 4.1. Connection without External Load

**Figure 4:**
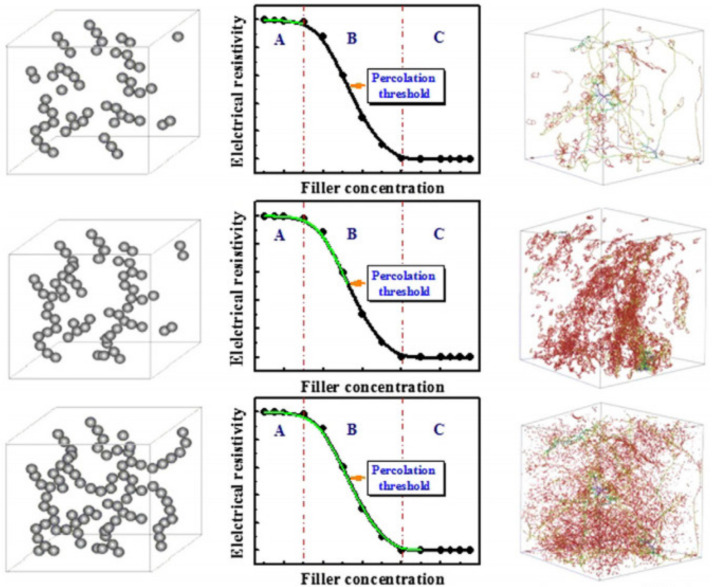
Variation of the electrical resistivity with change of functional filler concentration. Reproduced with permission from [44], Elsevier, 2014.

The concentration of functional fillers inside of the concrete matrix influences the change of electrical resistivity. The relationship between them is demonstrated in Figure 4. From the curve, the percolation phenomenon can be observed, and its presence is described by different concentration of functional fillers [42,43]. The curve can be divided into three parts: zone A with the high value of resistivity, zone B with a sudden decrease in the resistivity and zone C with low resistivity. 

Zone A is called the insulation zone, and it is characterized by a low concentration of fillers and large spacing between them. The large distance between fillers results in the problematic formation of the conductive path, which creates difficulties for the electron’s movement and causes lower conductivity of SSC. In this region, the ionic conduction prevails in the concrete matrix [4].

Zone B, which is the percolation zone, contains an increasing concentration of the fillers in the concrete matrix, thus providing smaller spacing for fillers and creating a better conductive path. Due to easier electronic transition, the conductivity of the sensing concrete increases. In the percolation zone below the percolation threshold, different types of conductions dominate, such as contacting conduction, tunneling conduction and ionic conduction. After the concentration of fillers surpasses the percolation threshold, the tunneling conduction and direct contact between functional fillers prevail in conductivity [44].

Zone C is a conductive zone that is described as a zone with a high concentration of the functional fillers, which mostly has total contact with each other; thus, the prevailing conduction in this region is the direct contact between fillers [4].

### 4.2. Connection with External Load

During the loading condition, the electrical resistivity of SSC changes, and this is caused due to the change of several factors: intrinsic resistance, bonding between concrete matrix and fillers, contact between fillers, tunneling distance and capacitance [4,45,46,47,48]. 

The mentioned factors could work together and have an impact on the sensing properties of concrete. In the insulation zone (Zone A), the conductivity of the concrete is very low, and it is difficult to create the conductive pathway there. Therefore, the self-sensing response in this zone is weak or completely absent during loading conditions due to high electrical resistivity. 

In the inchoate of zone B, the factors such as change of capacitance, intrinsic resistance of fillers and the change of bonding between concrete matrix and functional fillers mainly prevail. Near the percolation threshold zone, the main influencing factor is the variation of tunneling distance, while the dominant factors are the change of contact between fillers, tunneling distance between fillers and intrinsic resistance of fillers at the end of zone. The sensitivity of the concrete is good in this zone due to many factors that affect the sensing properties [42].

In the conductive zone, C, the leading factors are the change of contact between fillers and the change of intrinsic resistance due to the dense packing of the fillers and their close contact. Consequently, in the composites, the change in sensitivity is hard to achieve, and low initial electrical resistivity also prevails here. As a result, in the formation of SSC, the percolation threshold is an important parameter because it represents the balance between low functional fillers concentration, high sensitivity and low electrical resistivity, thus helping to find an optimum design of the concrete [49]. 

## 5. Sensing Properties of Self-Sensing Concrete

The relationship between change in electrical resistivity and external force can characterize the sensing behavior of concrete. Additionally, the stress sensitivity coefficients, amplitude of change in electrical resistance and force sensitivity coefficients are other factors that can describe such an important parameter as concrete sensitivity. Overall, the main concept of self-sensing is that concrete can demonstrate different sensing behaviors at various loading types, such as compression, tension and flexure, and it can be affected by many factors that have an impact on the electrical resistivity of the composites, which is described further.

### 5.1. Self-Sensing Concrete under Different Loading Conditions

#### 5.1.1. Behavior under Compression

**Figure 5:**
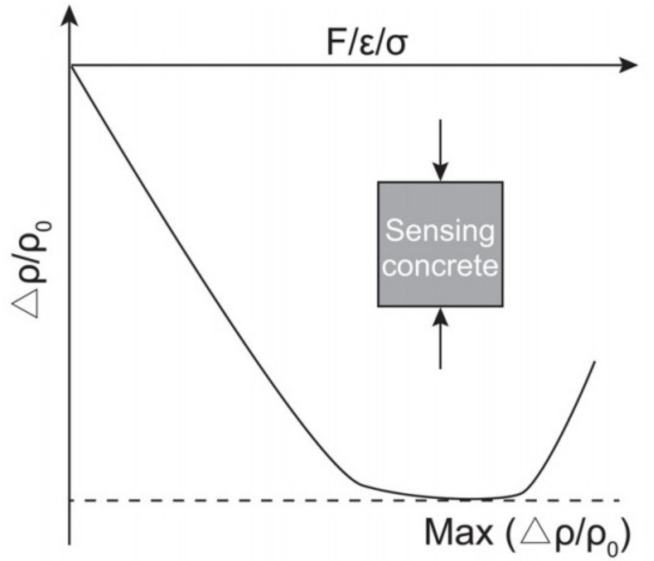
Self-sensing concrete under monotonic. Reproduced with permission from [51], Journal of Applied Physics, 2019.

Different behavior of SSC can be observed at various loading types [50]. During monotonic compression, with an increase in load, the values for the electrical resistivity $\Delta \rho /\rho_{0}$ firstly decrease, then become balanced and after that suddenly increased, as shown in Figure 5. Such a sequence of states is explained by the compaction pressure, creation of fresh cracks and then their expansion [21]. Firstly, under monotonous uniaxial compression, the distance of conductive particles decreases, thereby improving the conductive network within concrete. Then, the destruction, as well as the reconstruction of this network, is observed during the development of new cracks. Finally, the expansion of cracks results in the disruption of the conductive network [51]. 

During the loading stage, when the sample is subjected to repeated compression, the electrical resistivity $\Delta \rho /\rho_{0}$ decreases but it again increases during the unloading stage. However, the electrical resistance at the beginning of each loading cycle is different compared to the resistivity at the initial condition of the specimen, which was not subjected to the compression [52]. The reason for different values is explained by the defects in the concrete such as internal holes or cracks that are reduced during the compression. Therefore, the initial value for the electrical resistivity is not similar to the baseline of the composite that was already loaded, and stress returned to zero. Additionally, the variation of the $\Delta \rho /\rho_{0}$ could be explained by the ultimate strength of SSC. During repeated compression, if the sample is subject to the amplitude of the stress equal to 30–75% of the ultimate strength, then the baseline resistivity will be irreversible and the change in electrical resistivity will be reversible, while at the amplitude lower than 30%, the baseline together with a change in electrical resistivity will be reversible [4]. The exception where both baseline and change in electrical resistivity are irreversible is at compression stress amplitude of more than 75% due to the destruction of the conductive network.

**Figure 6:**
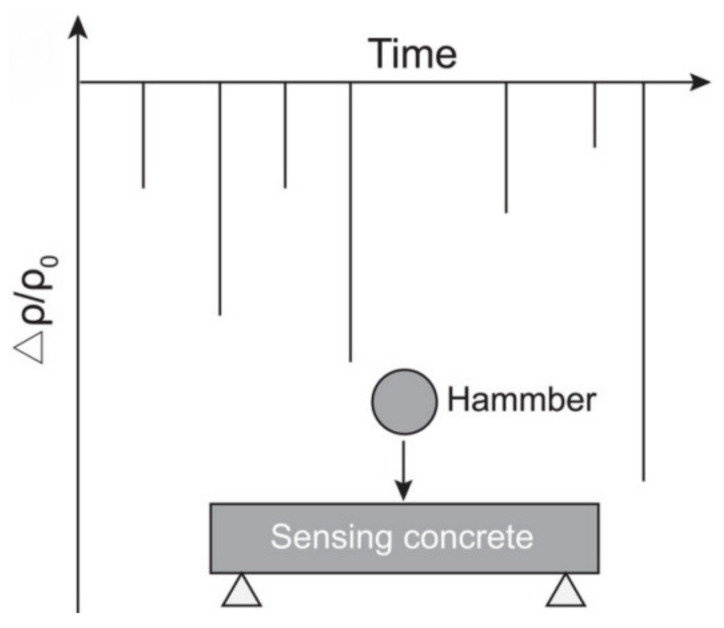
Self-sensing concrete under impact load. Reproduced with permission from [51], Journal of Applied Physics, 2019.

One of the most common loads to which concrete structures are subjected to is an impact load. During impact loading, $\Delta \rho /\rho_{0}$ decreases abruptly but then returns to 0 after loading, as it can be observed in Figure 6. The electrical resistance of the sample depends on the amplitude, i.e., the higher amplitude of the impact load, the higher the change in electrical resistance will be [52]. According to Meehan et al. [53], the high value of an impact load amplitude or a large number of impacts can result in the state where electrical resistance will not recover and return to 0 due to the inflicted damage inside of SSC. However, very few researchers have investigated the behavior of SSC subjected to the impact load. Hence, more research should be conducted for a better understanding of the response mechanism under impact loading [4].

#### 5.1.2. Behavior under Tension

**Figure 7:**
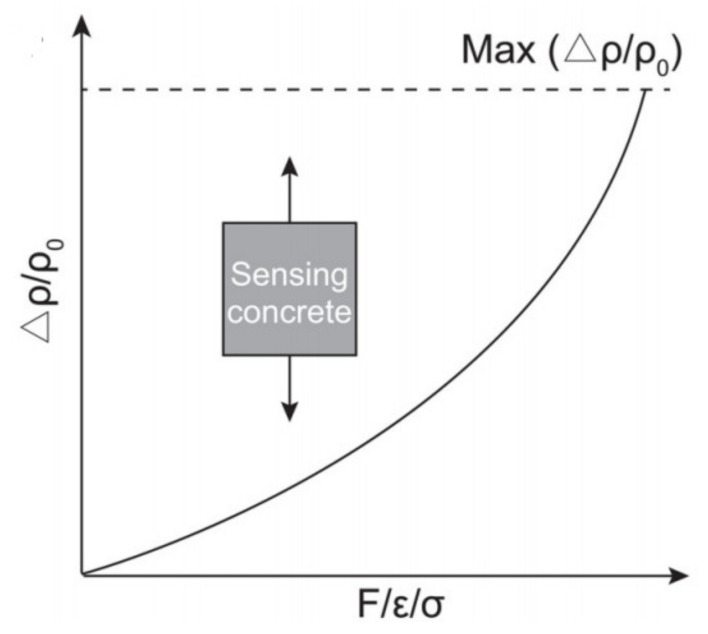
Self-sensing concrete under monotonic tension. Reproduced with permission from [51], Journal of Applied Physics, 2019.

During the monotonic tension, the electrical resistivity $\Delta \rho /\rho_{0}$ increases with the increase in tensile stress as the fillers that are inside of the concrete separate, and the microcracks are created (Figure 7). However, the fractional change in electrical resistance starts to increase much faster after the ultimate tensile strain because of the damage formation [52]. Therefore, sensing of the concrete depends not only on the tensile strain but also on the cracking behavior inside of the sample [51]. Reza et al. [54] studied the self-sensing behavior of concrete with CF under compact tension. The researchers found out that the electrical resistance can be used to observe the mechanisms of the fracture process zone and, thus, help to calculate the propagating crack length.

When the sample is subjected to the repeated tension, the electrical resistivity increases with loading, but the electrical resistivity decreases during the unloading phase. The behavior of the change in the $\Delta \rho /\rho_{0}$ is similar to the loading under repeated compression where the baseline and the electrical resistivity are different at different values of the amplitude [4]. In particular, within the elastic region, the baseline and the electrical resistivity changes are reversible, while both of them are irreversible when the elastic deformation range is exceeded by loading amplitude.

#### 5.1.3. Behavior under Flexure

**Figure 8:**
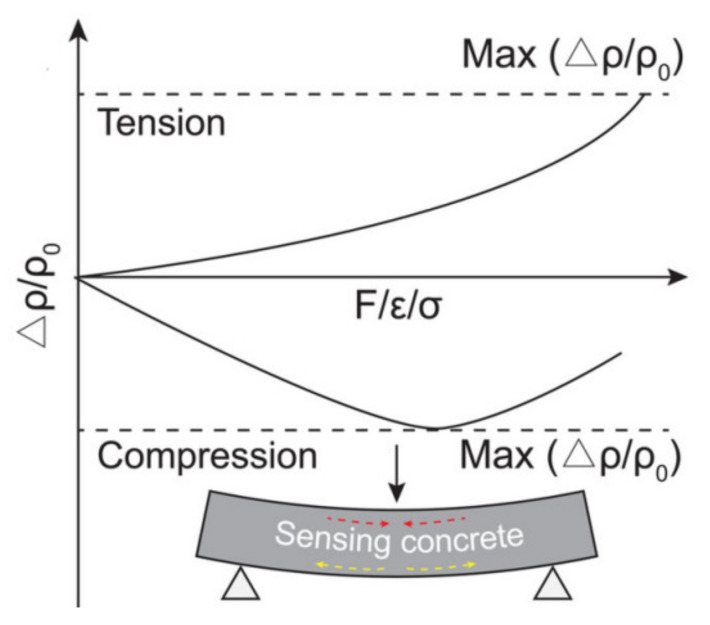
Self-sensing concrete under flexure. Reproduced with permission from [51], Journal of Applied Physics, 2019.

Tension and the compression parts of SSC work in the opposite way when the beam is subjected to the load at the center [4], as can be seen from Figure 8. Under the deflection of the concrete sample, the sensitivity of the tension side is much greater compared to the compression, which can be explained by the high compressive strength of concrete in contrast to its tensile strength [7]. 

According to the research study conducted by Wang et al. [55], the electrical resistivity reached the lowest point at ultimate flexural strength as load increases, then a sudden increase in electrical resistivity was noticed after increase in loading. Additionally, research conducted by Azhari and Banthia [26] showed that in a sample with 5 vol% of CF, firstly, the electrical resistivity decreased suddenly with the increase in the vertical displacement of the concrete. However, when the displacement became 0.3 mm, the resistivity started to decrease gradually and stopped at the 0.35 mm displacement. It was also observed that after 0.35 mm displacement, the electrical resistivity started to increase with the rise in loading until the failure of the sample. In another research conducted by Chen [56], the author compared the change in electrical resistivity of samples prepared with 0.22, 0.55 and 0.80 vol% of CF. The results showed that in the specimen with 0.55 vol% of CF, the electrical resistivity decreased with the increase in loading; however, contrary results were obtained for samples prepared with 0.22 and 0.80 vol% of CF, where the resistivity increased with the increase in loading. Overall, the results of different researchers showed that electrical resistivity can be used as one of the factors for monitoring the cracks, damage extent and the stress and strain of SSC.

### 5.2. Factors Influencing Electrical Resistivity: Functional Filler Concentration, Geometric Shape of Functional Fillers, Temperature, Loading Rate, Moisture and Some Other Factors

**Figure 9:**
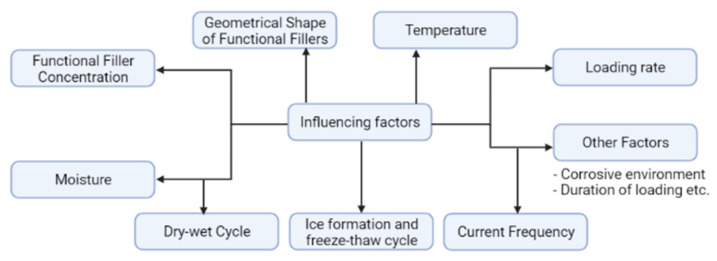
Factors influencing the electrical resistivity.

Electrical resistivity can be influenced by many factors, including the external forces, components of the concrete, technology used and different environmental conditions (Figure 9) [4]. The following section includes information about different factors that affect the electrical resistivity of SSC. 

#### 5.2.1. Functional Filler Concentration

The concentration of the fillers in the composite affects the distribution and also a formation of the conductive network; hence, it is one of the most important parameters that influence the properties of SSC [57]. The dependence of the sensitivity of the electrical conductivity of the concrete on the amount of the fibers was investigated by Wang et al. [55] under a three-point bending method, thus evaluating the effect of the content of carbon fibers on the self-sensing behavior of the composite. Similarly, Lin et al. [58] examined the sensing behavior of cement composites with a concentration of CB equal to 0.25%, 0.5% and 1% by weight ratio to total cementitious content. The authors found that the composites with increased CB content mostly had an increase in the gage factors in both states, elastic and inelastic. Chen et al. [59] compared and analyzed the sensing properties of mortar with different concentrations of carbon fibers under compression. Results from the experiment indicate that there is firstly an increase in sensitivity and then a decrease during the addition of more fibers. It was demonstrated that a certain value of volume fraction higher than the threshold will not increase the change of electrical conductivity. This phenomenon can be explained by percolation theory, which corresponds to the effect of random changes on the quantity and quality of existing connected fibers [43]. Hence, desired sensing properties of concrete could be obtained using the proper volume fraction of functional fillers in the mix [27].

#### 5.2.2. The Geometrical Shape of Functional Fillers

The geometrical shape of fillers, including parameters such as structure, size and surface characteristics, also impacts the sensing properties of the concrete as it affects the conductive network. For instance, long carbon fibers can create a more continuous network, thus providing lower resistivity of cement composite but can negatively influence the workability and uniform distribution of fibers.

**Figure 10:**
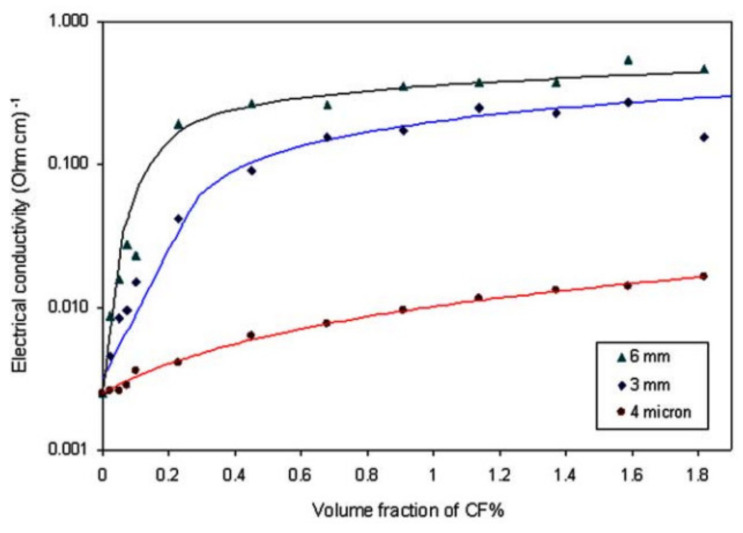
Impact of the fiber length on the sample in CFRC. Reproduced with permission from [39], Elsevier, 2005.

Chiarello and Zinno [39] evaluated the effect of different carbon fiber lengths (6 mm, 3 mm and 4 µm) on the electrical conductivity of a mortar. From the results presented in Figure 10, it can be noted that for the constant volume fraction of CF, the electrical conductivity increased with the increase in the length of the fiber. The data showed that with the increase in the volume fraction of CF (0 to 0.4%), the long fibers showed a sharp increase in electrical conductivity while the samples prepared with short fibers showed a gradual increase. From the observed effect with shorter fibers, the percolation threshold was at higher volume fractions. Thus, the authors concluded that with the optimum fiber length, the lower amount of fibers is needed to create a conductive network that can reach the conductivity threshold without negatively influencing the workability of the concrete.

Han et al. [30] evaluated the self-sensing properties of cement paste with various shapes of nickel powder (smooth spherical and spiky spherical). According to the results, the sensing property of smooth spherical nickel powder was weak compared to the strong sensing property of the spiky spherical nickel powder. Such findings suggested that the sharp nano angles of these particles can form tunneling effects as well as field emissions that have a very sensitive responses to compression. Overall, the geometrical shape of functional fillers should be selected accurately due to its influence on the distribution of the conductive network and mechanism.

#### 5.2.3. Temperature

**Figure 11:**
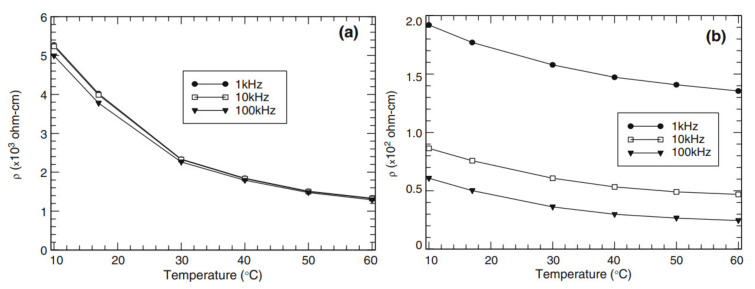
Variation in resistivity with temperature (**a**) plain cement mortar and (**b**) cement mortar incorporated with 3 mm carbon fibers. Reproduced with permission from [57], McCarter et al., 2007.

The change in the temperature results in the expansion or contraction of the concrete, thus impacting the distance between functional fillers, which in turn, influence the conductive network. Few researchers have evaluated the effect of temperature on the sensing behavior and the electrical resistivity of SSC. McCarter et al. [57] investigated the impact of the temperature from 10 °C to 60 °C on the plain cement mortar and the cement mortar containing carbon fibers. The results presented in Figure 11a,b show that the resistivity of both mixtures declined with the rise in temperature. The addition of carbon fibers (0.5% by volume) to the specimen made it less sensitive to the variation of the temperature compared to the plain mortar. From the obtained results, it was concluded that the increase in temperature is inversely proportional to the resistivity, i.e., with the increase in temperature, there is a decrease in resistivity. The same correlation was found by the researchers Li and Ou [60] and Teomete et al. [61]. According to Chacko et al. [62], the impact of the temperature is reversible, i.e., when temperature decreases, the electrical resistivity of the concrete increases and vice versa. 

#### 5.2.4. Loading Rate

**Figure 12:**
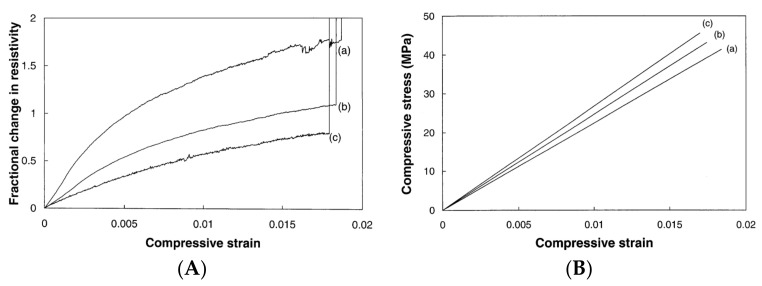
Strain during compressive testing up to failure at loading rates of (a) 0.144, (b) 0.216 and (c) 0.575 MPa/s versus (**A**) fractional change in resistivity and (**B**) stress. Reproduced with permission from [63], Elsevier, 2002.

The propagation of cracks in cement composites can be limited by a high loading rate, which can also suppress plastic deformation [52]. This could result in the changing trend of the conductive network during the loading state and, therefore, influence the properties of SSC. Cao and Chung [63] investigated the relationship between loading rate and the sensing behavior of cement mortar with carbon fibers under compressive load at loading rates of 0.144, 0.216 and 0.575 MPa/s. Figure 12A,B shows that the electrical resistivity increased with the increase in compressive strain/stress until failure. In addition, rise in the strain rate resulted in a decrease in the resistivity at any strain level, which resulted in a decrease in the resistivity at failure. More specifically, the load caused a constant change in the microstructure of the cement mortar and was mainly considerable at the inchoate stage.

Han et al. [64] evaluated the effect of loading rates (0.05, 0.10, 0.15, 0.20, 0.25 and 0.30 cm/min) on the sensing property of carbon nanotube cement-based composite under compressive loading. From the obtained results, it was determined that there was almost no effect on the sensing behavior below 0.20 cm/min. However, when the loading rate exceeded 0.20 cm/min, there was an increase in self-sensing response, and it was more significant with higher loading rates. Therefore, there is a dependence between different loading rates under compression with the sensing properties of SSC. 

#### 5.2.5. Moisture

**Figure 13:**
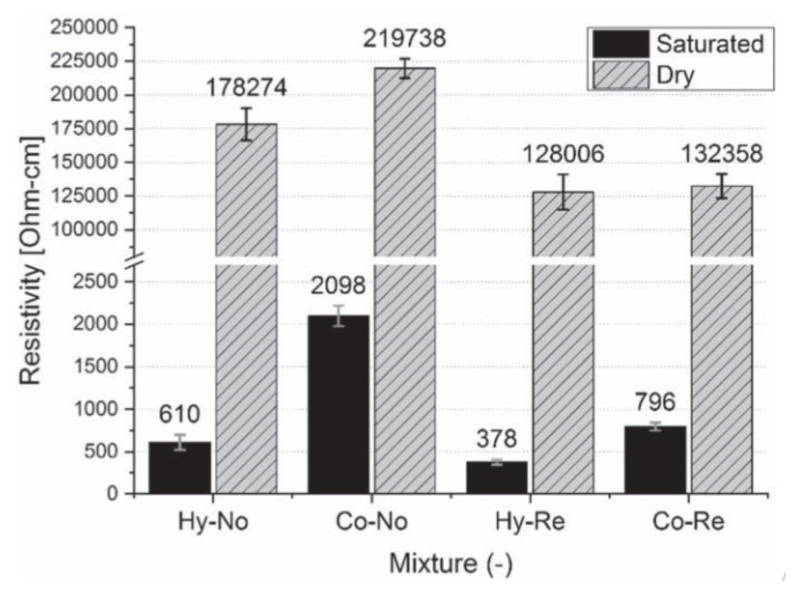
Resistivity of saturated and dry specimens. Reproduced with permission from [65], IOP Publishing Ltd., 2020.

Moisture content also influences the electrical resistance of sensing concrete. Very few researchers have evaluated the influence of moisture within SSC. Maier [65] investigated the impact of the moisture content on the hybrid fiber-reinforced concrete (HyFRC) that was incorporated with steel and polyvinyl alcohol (PVA) fibers in an amount of 1.3 vol% and 0.2 vol%, respectively. The results were compared with the control mix without rebar (Co-No), control mix with rebar (Co-Re), HyFRC mixture without rebar (Hy-No) and HyFRC mixture with rebar (Hy-Re) in both saturated and dry states (Figure 13). The difference in initial impedance values for all specimens can be observed regardless of whether the reinforcement was used or not. Dry samples showed higher resistivity compared to saturated specimens. Furthermore, the HyFRC sample demonstrated lower electrical resistivity than the control mix, which is explained by the presence of steel fibers. The presented results illustrate the influence of the moisture content on electrical resistivity due to the change in the electrical conductivity of fillers and concrete matrix. Overall, the influence of the moisture content can help to show the realistic environmental conditions under which SSC will be subjected [26].

#### 5.2.6. Dry-Wet Cycle

It is known that generally, in the dry state, conduction by electrons is dominant, while the ionic conduction is more significant than electronic conduction in the wet state due to the presence of ions inside of the water. Wen and Chung [66] investigated the influence of the dry-wet cycle on the electronic conduction in carbon fiber reinforced cement. For this purpose, the authors used four samples of cement paste with untreated carbon fiber and latex, ozone treated carbon fiber and latex, untreated carbon fiber and silica fume and ozone treated carbon fiber and silica fume. The authors also examined the effect of the latex and silica fume admixtures on the sample’s conductivity. It was determined that, with the presence of the latex admixture, the wet ionic conductivity was considerably higher compared to the dry overall conductivity. By contrast, silica fume provided higher electronic conductivity comparable to wet ionic conductivity. Overall, it can be concluded that the resistance and conductivity of the self-sensing sample change with the water content under the dry-wet cycles, which is mainly because of the polarization [67]. It results in a reduction in durability, which is explained by unstable water content inside of the concrete. This, in turn, further destroys the conductive network as well as the sensing property of the concrete matrix [4].

#### 5.2.7. Ice Formation and Freeze-Thaw Cycle

**Figure 14:**
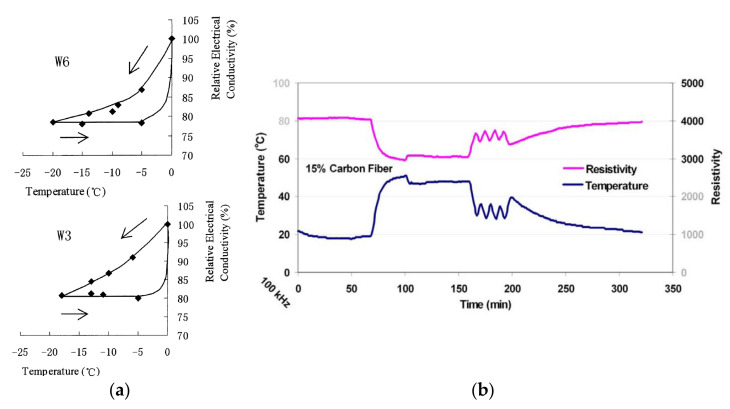
(**a**) Below zero temperature variation; (**b**) above zero temperature variation. Reproduced with permission from [68], Elsevier, 1998. Reproduced with permission from [69], University of British Columbia, 2008.

The ice formation and the freeze-thaw cycle can affect the electric conductivity and electrical resistivity, which in turn degrade the properties of SSC. For instance, Cai and Liu [68] evaluated the effect of ice formation on the concrete electric conductivity for conventional, high strength and concrete pore solution. Specimens that were used during the experiment were cured for 3 months and were cut into rectangular shapes with the size of 100 × 100 × 30 mm. According to Figure 14a, the data collected from unreinforced sensing concrete samples with w/c ratio of 0.3 (W3) and 0.6 (W6) demonstrated the loss of electrical conductivity during one cycle of temperature variation from −20 °C to 0 °C, thus providing poor repeatability. By contrast, the research presented by Azhari et al. [69] evaluated the electrical conductivity of the concrete sample with 15 vol% CF without the freezing cycle ranging temperature from 20 °C to 50 °C. As shown in Figure 14b, after one cycle of temperature variation, the electrical conductivity of the cement-based sensor increased with the decrease in temperature.

**Figure 15:**
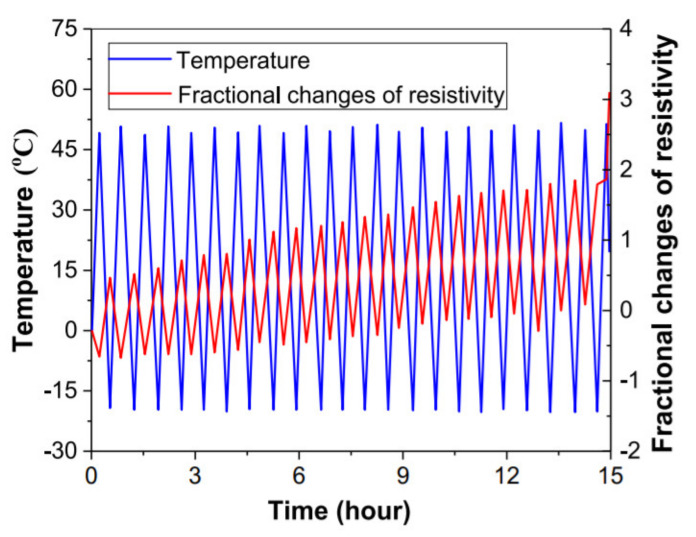
The fractional change in resistivity during temperature variation. Reproduced with permission from [70], Elsevier, 2002.

The influence of the freeze-thaw cycle on cement mortar was studied in detail by Cao and Chung [70], who compared the fractional changes of resistivity with a temperature range from −20 °C to 52 °C. During the experiment, it was identified that less damage was caused during the heating process in contrast to the freezing process. Therefore, it is evident that ice formation and melting processes are responsible for severe damage and low repeatability of fractional change of resistivity, as shown in Figure 15. Overall, a change in temperature during ice formation reduces conductivity along with the freeze-thaw cycle, which in turn results in severe damage of self-sensing properties. 

#### 5.2.8. Current Frequency

**Figure 16:**
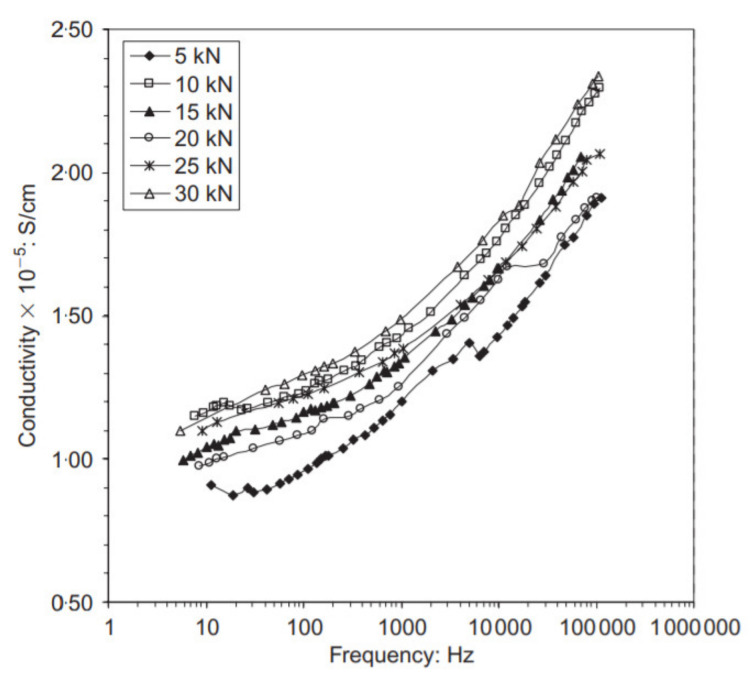
Change in conductivity versus frequency during loading process. Reproduced with permission from [71], Demirel et al., 2006.

The current frequency, which is used to measure changes before and after different loading conditions, is another factor that has an influence on the self-sensing properties of the concrete. Demirel et al. [71] analyzed the impact of the applied current frequency on SSC behavior containing carbon fibers. It was found that regardless of the magnitude of loading, the conductivity of CFRC under compression increased with the increase in current frequency, as shown in Figure 16. Therefore, the result of the experiment suggested that the effect from the current frequency is independent of the magnitude of loading.

#### 5.2.9. Other Factors

Apart from the factors discussed above, some other factors include corrosive environment and duration of loading, etc. [27,52,71], which affect the self-sensing properties of concrete. For example, the corrosive environment is considered to be one of the factors that can influence the properties of the concrete. Strong acids and alkali together with a high concentration of sulfate and chloride can reduce the electrical resistivity because of the ion permeabilization which, in turn, destabilizes the sensing properties of concrete [4]. However, the destruction of the conductive network inside is expected if there is a long-term contact with a corrosive environment that will cause degradation of SSC property. Research performed by Li et al. [67] investigated the effect of the long-term loading on the carbon black cement composite (CBCC) encapsulated with epoxy. The results obtained from the experiment demonstrated that the creep behavior was noted, but overall the resistance of CBCC specimen was not influenced by long-term loading. In conclusion, many factors affect the sensing properties of concrete and influence its performance; hence, for the sake of better observation and understanding of its properties, it is suggested to monitor and consider them in real-life applications.

## 6. Self-Sensing Materials for Smart Composites

There are many research studies conducted with functional fillers in SSC. The following section focuses on the research conducted on self-sensing materials used for smart composites, which are listed in Table 4. 

### 6.1. Carbon Fibers

**Figure 17:**
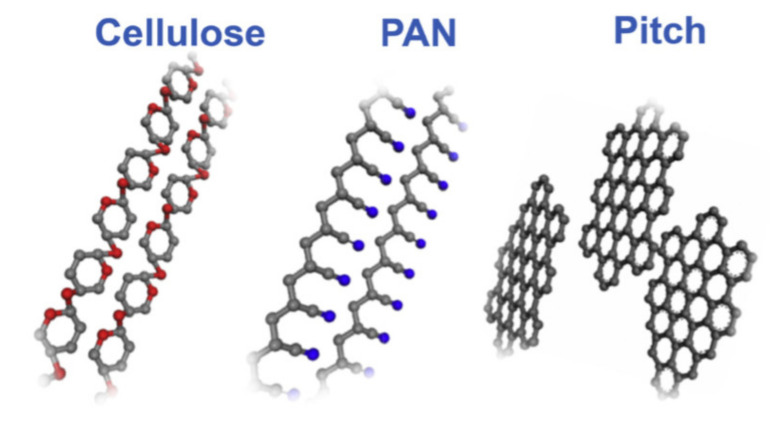
Structures of different precursors of carbon fibers. Reproduced with permission from [80], Elsevier, 2015.

Carbon fibers that are implemented in the concrete could be divided into several types. The majority of the carbon fiber market uses polyacrylonitrile (PAN) carbon fibers, the next priority is given to pitch carbon fibers and lastly to rayon carbon fiber textiles [79]. The implementation of different precursors of carbon fibers such as pitch-based (short) fibers, PAN-based (continuous) fibers and cellulosic carbon fibers provides different properties (Figure 17) [80]. Short and continuous carbon fibers, including carbon fiber-reinforced polymer and its hybrid composite, are discussed in detail in the next sections.

#### 6.1.1. Properties of Carbon Short Fiber-Based Self-Sensing Concrete

Short fibers are mainly used in concrete to increase the flexural toughness and strength and, at the same time, to decrease drying shrinkage [40,81]. Similarly, the carbon short fibers (CSF) that are pitch-based are used as admixtures in cement-based materials for mechanical performance improvement and damage monitoring [81]. 

**Figure 18:**
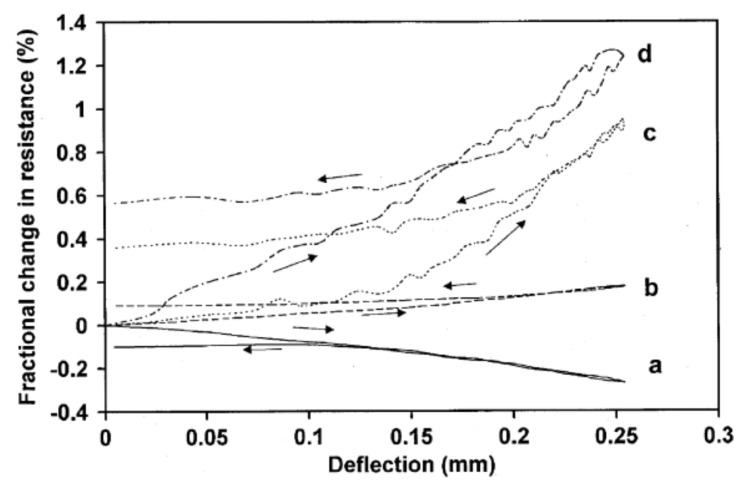
Variation of fractional change in resistance versus deflection at loading and unloading stage in the 1st loading cycle: (a) compression side surface resistance; (b) through-thickness resistance; (c) tensile side surface resistance; and (d) oblique resistance. Reproduced with permission from [40], Elsevier, 2006.

Several researchers evaluated the performance of carbon fibers including Wen and Chung [40], who specifically evaluated carbon fibers (diameter 15 µm and length 5 mm) in the amount of 0.5% by mass of cement by comparing the fractional change in resistance with the deflection at stages of loading and unloading. Type I Portland cement was used, and the fibers were dispersed inside of the cement by using a rotary mixer. Silver paint and copper wires were used as electrical contacts to measure values and were installed on the tension and compression side of the beam. The results of the comparison of (a) compressive side surface resistance; (b) through-thickness resistance; (c) tensile side surface resistance; (d) and oblique resistance presented in Figure 18 demonstrated that the biggest change was observed for the oblique resistance. It can also be observed that the resistance under unloading is higher than when loaded at the deflection below 0.17 mm, while the resistance in the loading phase is higher than the unloading phase above 0.17 mm deflection. According to the authors, initially, the deformation dominates in the unloading stage, which is further followed by an irreversible effect caused by damage in the next unloading stage. Therefore, it was concluded that both strain and damage have an impact on the change in resistance of the specimen.

**Figure 19:**
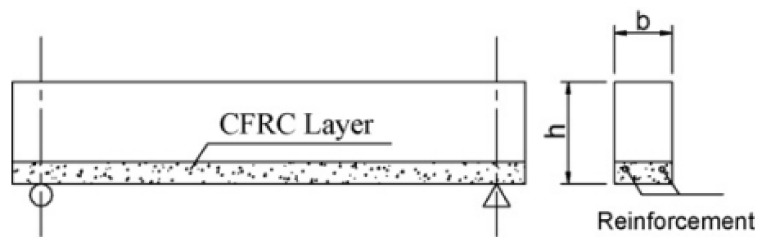
CFRC strengthened RC beam design. Reproduced with permission from [72], Elsevier, 2008.

Wang et al. [72] evaluated the influence of the thickness of the CFRC layer on the self-sensing capability of the beam. The authors used CSFs with a length of 5 mm in the reinforced concrete (RC) beam for fatigue damage control and sensing by designing the CFRC-strengthened part at the bottom layer of the beam, as illustrated in Figure 19. Four electrodes made from stirrups were located in the mid-span of the beam in parallel and had an interval of 200 mm between each other. Those electrodes were stuck out from the bottom of the beam for electrical resistance and the fractional change measurement. 

**Figure 20:**
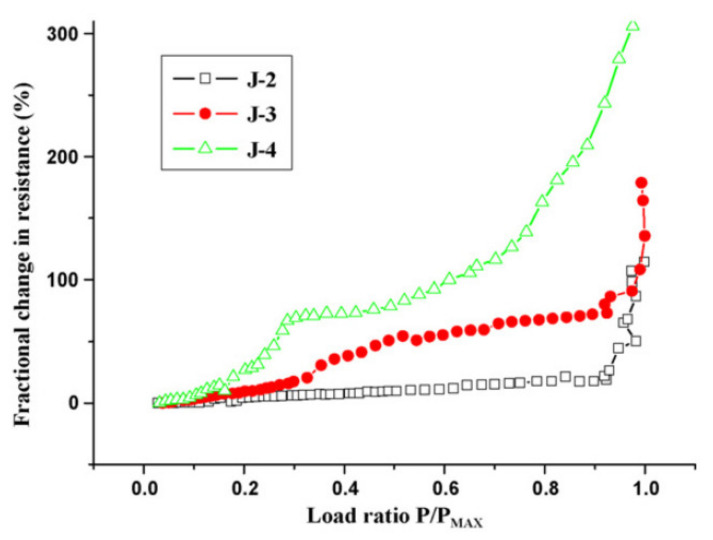
Variation of fractional change in electrical resistance versus load ratio for various CFRC layer thickness: J-2 (30 mm), J-3 (60mm) and J-4 (90 mm). Reproduced with permission from [72], Elsevier, 2008.

The results of fractional change in resistance with a load applied monotonically on this RC beam are presented in Figure 20. It is clear that, for all CFRC layer thicknesses, the fractional change in electric resistance increased with the increase in the flexural loading. At lower loads, small damage in the form of micro-cracks was noticed, which further continued to increase in size and width with the increase in loading. Minor damages resulted in the slow and stable development of fractional change in resistance caused by a disturbance in the conductive network between carbon fibers. However, with increasing monotonic loading until the failure load, severe damage to the beam was observed. The formation of continuous cracks reduced the signal transition between fibers and resulted in severe destruction of the conductive network. The effect of the damage can clearly be observed in Figure 20, where a considerable and abrupt increase in the fractional change in the resistance emerged. 

**Figure 21:**
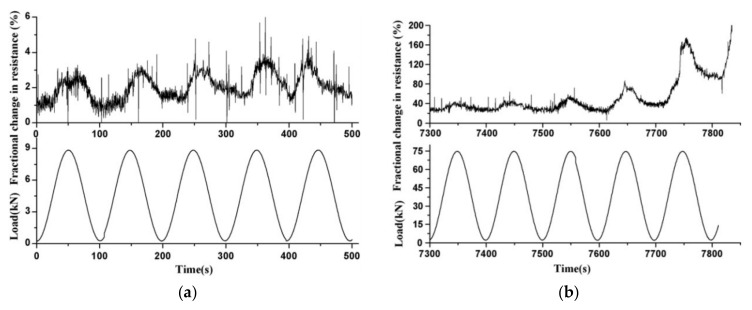
The fractional variation in electrical resistance versus time during repeated flexural loading: (**a**) at first 5 cycles; (**b**) at last 5 cycles. Reproduced with permission from [72], Elsevier, 2008.

The authors also compared the cyclic flexural loading effect on the electrical resistance for the first and last five cycles, the results of which are presented in Figure 21. The fractural change in resistance at the stress amplitude equivalent to 80% of first cracking stress is demonstrated in Figure 21a. In the first five cycles, the increase in loading affected the opening of the crack, thus causing an increase in the fractural resistance values. By contrast, the resistance decreased during the unloading stage due to crack closure. In addition, during the first five cycles, a minor increase in the fractional change in electrical resistance was observed from 2.8% to 3.6%. The insignificant damage caused to the beam during the 50 cycles resulted in a less than 7% increase in fractional resistance. Contrarily, during the last five cycles and at the stress amplitude equivalent to 80% of the ultimate stress, there was a dramatic increase in fractional resistance with irreversible effect before the failure of the sample, which took place in a total of 38 cycles. The fractional change in resistance reached a net increase value of 179%, as illustrated in Figure 21b. Therefore, the residual resistance, which represents an increase in the value of electrical resistance, can be used as one of the parameters for better control of fatigue damage in reinforced concrete beams.

Carbon fibers dispersed in a cement-based material can have great potential for fatigue damage monitoring in different concrete structures. Based on the literature reviewed above [40,72] and the other research studies conducted by [7,38,74], the influence of the strain and damage on the fractional change in the resistance of the beam was confirmed, and the use of this information could further help to monitor the structural condition of different concrete elements.

#### 6.1.2. Properties of Carbon Continuous Fiber-Based Self-Sensing Concrete

##### Carbon Fiber Reinforced Polymer and Its Hybrid Composites 

CFRP are polymer matrix composite materials mainly reinforced by carbon fibers. CFRP are classified as thermosets such as epoxy and polyester; and thermoplastics such as nylon that are manufactured by open and closed mold processes as well as with the pultrusion method. Different carbon hybrid composites and other fibers such as glass and aramid are designed for strength and sensing efficiency improvement [81]. The dispersed phase can be in the form of continuous or discontinuous carbon fibers typically in a diameter of 7 µm and woven in a cloth [82]. Mechanical properties of polymer composites rely upon several variables such as orientation, type of using fibers and architecture of the fiber. Specifically, architecture refers to the configuration of preformed textile, which differs by braiding, knitting and weaving. 

Overall, polymer composites are anisotropic materials and exhibit piezoresistivity under various loading types. For instance, the unidirectional CF reinforced composites with epoxy were found to sense their strain along the fiber direction. These continuous carbon fiber composites have highly desirable qualities such as high strength, material toughness, fatigue endurance, lightweight, resistance to abrasion, corrosion and chemical attack [83]. However, the main disadvantages of carbon fiber/epoxy composite are their high price and low tensile failure strain due to the intrinsic brittleness [84]. Therefore, to achieve the desired mechanical properties for the ductile reinforcement, different types of hybrid composites have been investigated [81]. These composites are mainly used because they are capable of generating the alarm signal before the failure of the structural element. The arrangements of fibers, as well as proportion and properties, are essential factors for pseudo ductility and self-sensing behavior.

**Figure 22:**
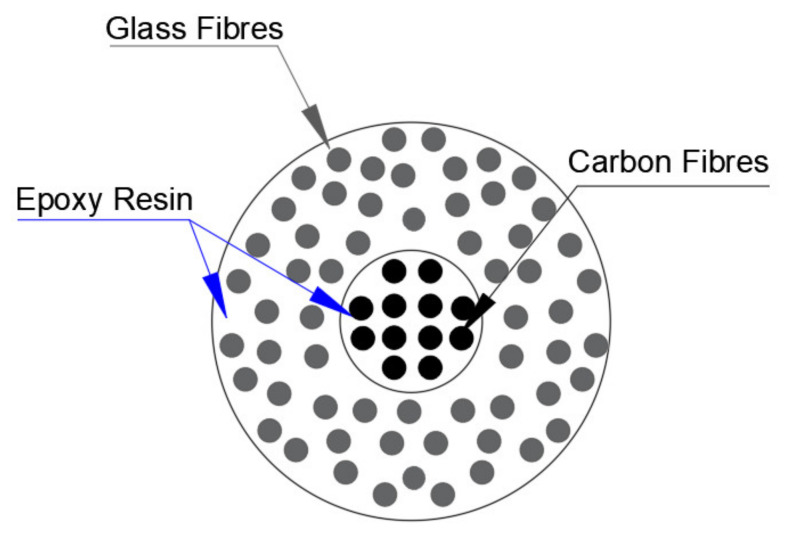
Schematic diagram of a CF–GFRP rod [73]. Modified for explanation.

One of the most commonly used hybrid composites is the carbon fiber-glass fiber (CF–GF) hybrid composites. The glass tows contribute as a reinforcement platform, while carbon fibers provide piezoresistivity, thus serving as the sensory agent [16]. From Figure 22, it can be noted that the internal conductive core of CF-GFRP rods is coated by coaxial GFRP skin [73]. The sensitive part, which is based on the carbon fibers, is wrapped around by glass fibers [73]. The investigation results showed that CF–GF hybrid composites demonstrated reliable sensing behavior under repeated cyclic and monotonic loading states. 

For electrical measurements, electrical contacts were installed at each end of the carbon rod through connection with copper electrodes. An abrupt increase in electrical resistance was observed in the specimen with CF during the monotonic tensile test. The reason for a sudden increment is the breakage of carbon fibers, which can be treated as an emergency or alarm signal. The particular value of a load before the alarm signal can be designed by using a variation of the proportion of glass and carbon fibers [73]. If the quantity of the CF–GF was higher (CF at 2.4% and GF at 49%), the warning signal would be produced just before the breaking load. Meanwhile, the usage of a lower quantity CF–GF (CF at 0.2% or 0.6% and GF at 48%) would indicate the increase in the electrical resistance much earlier than the breaking load, thus increasing the resistance at the values lower than at breaking load. Therefore, promising applications of hybrid composites in civil engineering structures were demonstrated.

**Figure 23:**
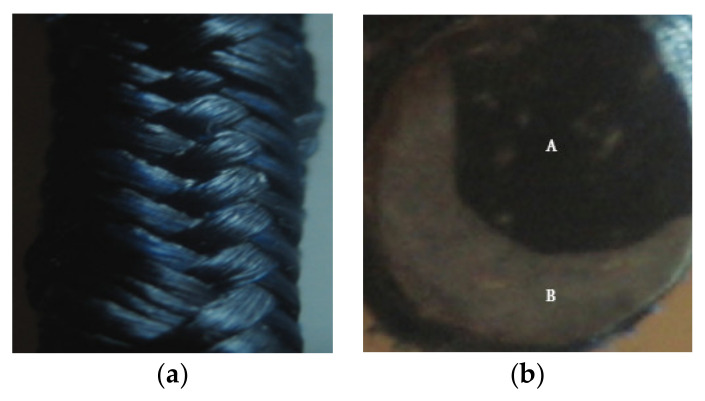
(**a**) Surface texture of BCR; (**b**) Cross-section of distributed carbon fiber: A—carbon fiber; B—matrix. Reproduced with permission from [85], Hindawi, 2014.

Nevertheless, the major limitation of hybrid CF–GF hybrid composites is their failure during early stage damage disclosure [50]. According to Rana et al. [81], the variation of the fractional resistance in the sample at low strain was very insignificant and better results could only be obtained in the pre-stressed condition. Therefore, to overcome this issue, different types of hybrid composites were tested, such as carbon fiber reinforced braided composite rods (BCRs), as shown in Figure 23 [85].

The yarns intertwining process is the main principle of braided composite rods. Polyester fibers are introduced for the braided structure while the glass fiber and the carbon fibers are combined for the core reinforcement development, thus producing BCRs [86]. The main feature of this method is that there is a certain change in the alignment of the axial carbon fibers, which is caused by the braiding of polyester yarns. Consequently, this misalignment of axial carbon fibers under a certain load and the appearing alteration in the electrical contacts cause a significant change in electrical resistance, including at low strain levels [85]. Controlling such braiding parameters such as tension and speed, etc., can help to regulate the degree of misalignment. Overall, BCRs in comparison with steel rebar, has higher tensile strength and lower modulus of elasticity.

**Figure 24:**
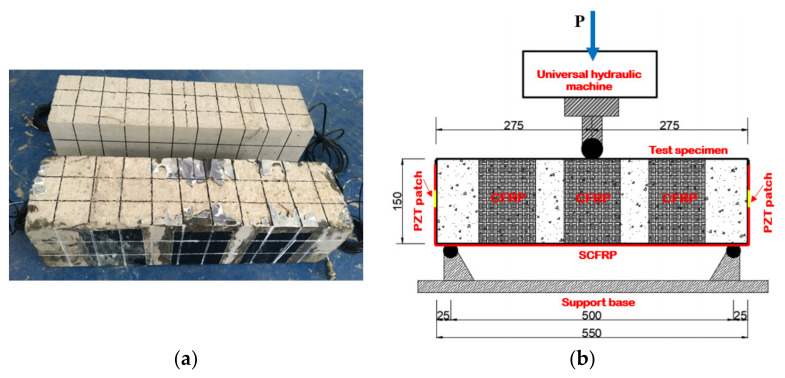
(**a**) Concrete beams with and without CFRP strengthening; (**b**) detailed loading setup. Reproduced with permission from [74], MDPI, 2018.

Additionally, there was a study conducted by Feng and Ou [74] that included concrete beams with different concrete grade (C25 and C40) and self-sensing carbon fiber reinforced polymer (SCFRP) as reinforcing material. Specimens with SCFRP and without SCFRP were analyzed, comparing their loading capacity and the displacement, as shown in Figure 24. In order to measure the signal, two PZT patches, which were located on the beam using a face-to-face configuration, were connected to a data acquisition board (NI-6363) and controlled by software. 

**Figure 25:**
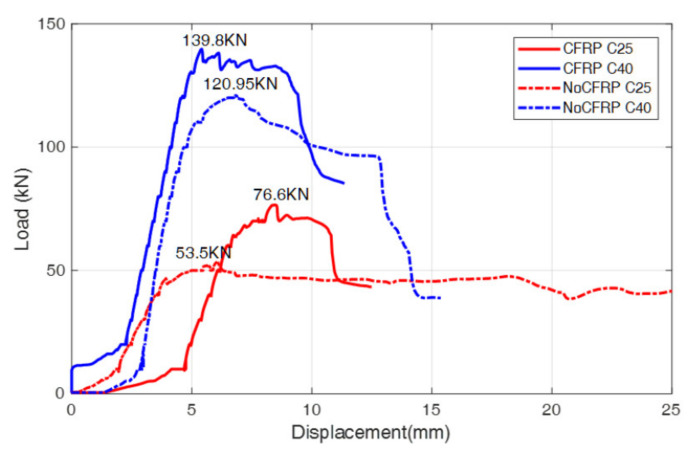
Load with displacement for different types of specimens. Reproduced with permission from [74], MDPI, 2018.

According to Figure 25, there is an apparent enhancement in the ultimate bearing capacity for different kinds of concrete grades, such as C25 and C40, with SCFRP fabric implementation. It was found that the concrete with a lower grade has a considerable increase in bearing capacity in comparison to the beam with a higher grade. The cause of that phenomenon is the difference in the failure mode. Additionally, the researchers found out that the destruction between the connection of CFRP fabric and C25 diminishes the effectiveness of CFRP in higher tensile or shear strength zones, whereas the beam with C40 concrete grade failed since CFRP fabric reached its ultimate bearing (Figure 25). Thus, implementing the appropriate layout and location of those fabrics is an important part of the concrete strengthening process. 

Finally, the study concluded that, with SCFRP fabric installment, the increase in bearing capability was observed, and the decrease in energy values was detected with the development of cracks in the beam. This suggests that the integration of SCFRP into concrete-based material has strength improving capabilities as well as an ability to monitor the development of damage in the concrete elements. Therefore, it can be inferred that CFRP has a promising future as the self-sensing technology for the health monitoring and strengthening of the buildings. 

##### Continuous Carbon Fiber 

**Figure 26:**
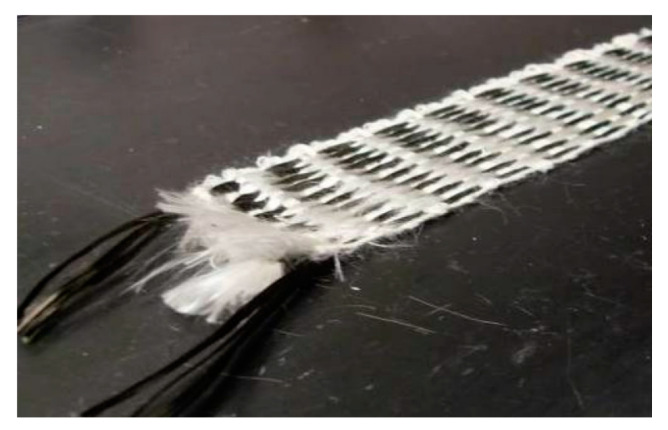
The image of carbon fiber textile. Reproduced with permission from [75], MDPI, 2015.

Continuous PAN-based carbon fibers were also developed for the strain and damage detection of the buildings [87]. The integration of these fibers into textile fabrics may be one of the methods for improving strength and self-sensing functions [88]. In one of the research studies conducted by Salvado et al. [75], a novel method of developing SCFRP fabric was presented. PAN-based continuous carbon fibers with unidirectional arrangement were used to create special polymer-matrix composites (Figure 26). 

**Figure 27:**
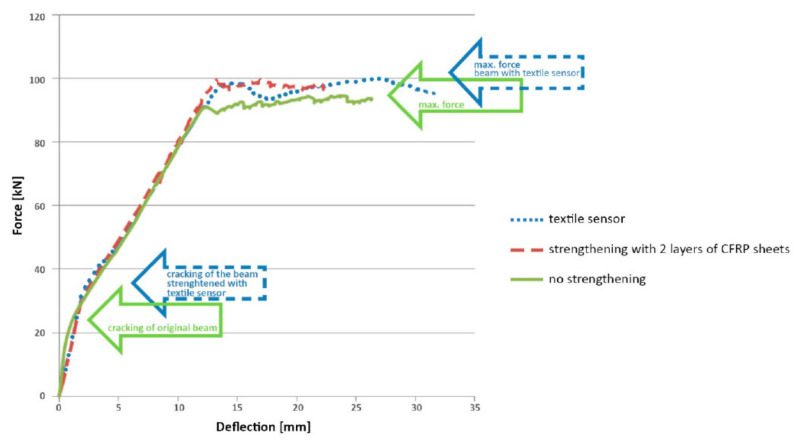
External load with a deflection for the 3 types of beams: without strengthening, with CFRP strengthening and with textile sensor strengthening. Reproduced with permission from [75], MDPI, 2015.

The experiment conducted by the research group used three different beam models without strengthening, with CFRP strengthening and with textile sensor strengthening. The data obtained in the calibrating test in beams showed the relationship of change of strain under external loading conditions, as presented in Figure 27. The graph demonstrated the effectiveness of the textile sensor in the specimen for strength improvement. According to the results, the final load carrying capacity of the beam with textile sensor increased by approximately 10%. The insufficient rise in load capacity can be attributed to the obtained result found by using short reinforcement anchoring, which resulted in low efficiency. However, the actual value may be higher with different lengths of reinforcement anchoring.

The total strength of the beam with strengthening was improved compared to the beam without strengthening, as evidenced by a significant increase in the force that caused the first cracks on the specimen. Similarly, it was observed that the small strain of the strengthened specimens caused an increase in the relative electrical resistance, which is explained by the misalignment of axial carbon fibers under load that resulted in decreasing numbers of connection points between fibers at different loading conditions, as previously mentioned. Therefore, the influence of the strengthening fabrics made with continuous carbon fibers could be used as self-sensors to improve the strength by increasing the loading capacity of concrete structure and also to perform the health monitoring by deformation detection that was caused by external loads [81]. 

Overall, it can be said that the carbon fibers, including short and continuous, can considerably improve the bearing capacity of the elements made of concrete and additionally can provide the ability for the reinforced concrete to monitor health conditions by self-sensing properties.

### 6.2. Carbon Nanomaterials

#### 6.2.1. Properties of Carbon Nanomaterials-Based Self-Sensing Concrete 

The development in the field of nanotechnology resulted in the implementation of carbon nanomaterials such as CNFs and CNT in self-sensing composite materials. CNTs are the most studied nanofillers in the literature that consists of graphene layers connected into a cylindrical shape, while CNF contains graphene layers in the shape of cones, plates or cups [81]. Both carbon nanomaterials show remarkable mechanical, thermal and electrical properties; therefore, this makes them a suitable material for casting concrete elements. Additionally, there are other carbon-based nanofiller such as CB and graphene nanoplatelets (GNPs) [89]. For SEM micrographs of nano-modified suspensions of nanofillers such as CNT, CNF, CB, and GNP, please refer to Figure 2 in Reference [76].

In a study conducted by Alessandro et al. [76], the authors evaluated the self-sensing ability of cement paste cubes made with different fillers such as CNTs, CNFs, CB and GNPs. CNTs had a diameter of 5–15 nm, and their length was approximately 500 µm. CNFs consisted of graphene plane surfaces mowed from 70 to 200 nm diameter fibers with length from 50 to 200 µm. CBs were spherically shaped carbon, while GNPs composed of stacks of graphene with 3–10 nm thickness. All of the cubic specimens had a water-cement ratio equal to 0.45. Inside of the cubes, five stainless steel nets were located at a regular interval of 10 mm (Refer to Figure 3 in Reference [76]). To take measurements, stabilized voltage was applied to two net electrodes using power source model PXI-4130, while the current between the electrodes was measured by an NI PXI-4071 high-speed digital multimeter. During the experiment, the applied load was monitored and the maximum value of the load that was reached during electromechanical cyclical tests was equal to 2 kN (Refer to Figure 6 in Reference [76] to see the graph). 

From test results, it was observed that CNTs and CBs demonstrated a polarization effect, which can be entirely eliminated after 20 min of charge without any external load. Additionally, it was indicated that the sensitivity of GNPs compared to other types of carbon fibers was the most uncertain and inaccurate and showed the worst results (Refer to Figure 7 in Reference [76]). Therefore, the suggestion that was made by the researchers regarding the implementation of GNPs is that a higher amount of concentration is needed in the cementitious matrix for a more effective self-sensing application. Overall, CNTs, CBs and CNFs presented linearity with strain. However, among all of the specimens, the cube with CNTs showed a good quality of signal and demonstrated higher conductivity. Similar conclusions regarding the effectiveness of the implementation of CNTs fiber for self-sensing ability were reported from the research studies of [24,28,36,41,89].

**Figure 28:**
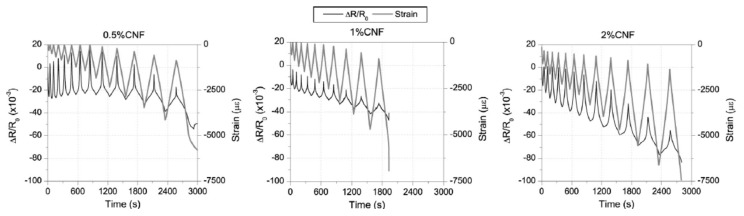
Relationship between resistance and strain with time for three types of cement pastes with 0.5%, 1% and 2% CNF concentration. Reproduced with permission from [10], Elsevier, 2014.

The addition of CNF in the cement matrix has been reported to provide a good self-sensing result. Recently, Galao et al. [10] evaluated the performance of CNFs for potential application in traffic monitoring and embedded CNT for damage detection. Firstly, the compressive strength of samples was compared with each other at different curing ages, and the improvement in strength after 28 days of curing was obtained. For this purpose, the authors evaluated the damage sensing performance of three different types of cement paste prepared with various concentrations of CNF (0.5%, 1% and 2%). Measurements were taken with a digital multimeter, KeithleyModel 2002, while an AC/DC current source, Keithley 6220, stabilized the electrical current intensity. During the damage-sensing test, the resistances of samples, as well as the strain, were monitored and compared, as shown in Figure 28. All the specimens were loaded until their failure, where ultimate stresses were 62.5, 49.8 and 58.6 MPa, respectively. The strain of the samples at failure varied from 0.5% (5000 με) to 0.8% (8000 με). After the damage introduction, all tested samples with CNF showed the non-linear behavior in the electrical response of the strain. Thus, there could be an irreversible modification in resistivity if the residual strain occurs. Overall, it can be observed that specimens with 2% of CNF fibers demonstrated the most accurate results for the strain and electrical resistance of the samples, among other reinforced cement pastes. 

The strain monitoring ability of CNF was studied by a lot of authors, including [10,32,35,90]. The findings of these research studies showed that, with the small strain, the response of the CNF showed good results; however, with the increase in strain value, the consequences after damage affected the self-sensing capability. Therefore, no correlation was detected between the change in electrical resistance and the strain of the specimen. Such a response indicates the presence of significant damage in concrete samples. 

Apart from conventional carbon fibers, the self-sensing properties of other types of nanomaterials, such as hybrid composite rods, have also been investigated. The research study conducted by Nanni et al. [77] developed the hybrid rod with an internal conductive core made of glass fiber included into the carbon nanoparticles (CnP)/epoxy mixture. The external insulating part was made of glass fibers reinforced polymers (GFRP) skin for mechanical performance improvement and electrical insulation, please refer to Figure 1 in Reference [77] to see the scheme and SEM micrograph. During the experiment, 5% concentration of CnP with an average 30 nm diameter was used. Measurements were made by applying a constant current while the voltage decay was evaluated using NI Field Point SG104 system. The results of the self-monitoring performance of the CnP-GFRP reinforced concrete beam showed that due to the application of the tensile load, the strain increased and caused the gap between fibers to increase, thus resulting in enhanced electrical resistance (Refer to Figure 7 in Reference [77]). It was concluded that the self-sensing of the hybrid composite was effective. 

**Figure 29:**
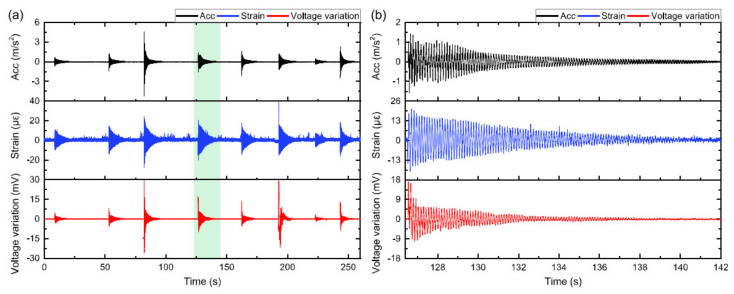
(**a**) Response of HNCS during the hammer impact test; (**b**) response-decaying curves for three sensors during the test. Reproduced with permission from [29], IOP Publishing Ltd., 2020.

Another relatively new hybrid nanocarbon materials engineered cement-based sensors (HNCSs) was proposed by Ding et al. [29]. The authors fabricated HNCS by using CNT and nano carbon black (NCB) composite fillers. At the optimal content of CNT/NCB equal to 6 wt% of cement, the best sensitivity was achieved. Figure 29a demonstrates the results from the hammer impact test with the recorded values during a specific period. 

It can be observed that three types of measuring sensors react almost simultaneously when the force was applied to the sample with only a little difference in the variation of impact intensities. From the graph of response-decaying curves of all three sensors that is shown in Figure 29b, it can be noted that the signal started to gradually decrease with time demonstrating a low noise level as well as a long time for decay, which indicates the potential usage of HNCS for damping estimation. Therefore, during the hammer test, the values of recorded results demonstrated consistency in the data, thus showing the future potential and capability of HNCS for its application in the structural health monitoring process. A similar conclusion was obtained by Han et al. [91], who also tested this material for its ability to monitor electrical resistivity and piezoresistivity of cement mortars. The authors identified that 2.40 vol% is the optimal content of CNT/NCB, which can be used for piezoresistivity [91]. The advantages of this technology are a relatively simple preparation procedure together with a stable and sensitive piezoresistivity even at a low content of functional fillers [92].

Overall, self-sensing hybrid composites have been developing for self-monitoring purpose, and a lot of recent research studies demonstrated high sensitivity during applied loads with such fillers. For example, Azhari and Banthis [26] evaluated the performance of 1 vol% of CNTs and 15 vol% of CF introduced into cementitious composites. Findings showed that both changes in loading and strain are similar to the variation in electrical resistance, thus presenting better results compared to the implementation of CF alone in the cementitious composites. Similarly, Ju et al. [93] investigated the self-sensing property of hybrid CNT-based multi-scale composite. The experiment results indicated that four different strain characteristics were found according to the change in the electrical resistance and were identified as critical strain levels. The capability of CNT-based multi-scale composite to be used to detect the micro-damage in the concrete structure was also shown by Wan and Guo [94]. Therefore, the feasibility of using carbon nanomaterials, including the hybrid composites for damage detection and self-monitoring, can be a perspective method for the structural health monitoring of the concrete elements. 

#### 6.2.2. Fiber Dispersion 

The conductivity of SSC can be improved by increasing the amount of the dispersed fibers; however, the role of their size and the distribution in the cement-based material should also be considered due to the ability to limit sensing performance. The conductive network, which is an essential factor for damage sensitivity, could be blocked by the poor dispersion of the fibers. One of the most practical ways to avoid high cost and impact on the workability of the concrete due to the increasing concentration of dispersed fibers is a uniform distribution of those fibers inside of cement-based material. One of the novel methods for dispersing the fibers was introduced by Gupta et al. [78], who suggested the spraying process of the CNT fibers on top of the aggregates (Refer to Figure 3 in Reference [78]).

The CNT-based thin films were applied on the surface of the fine (sand) and coarse aggregates before the concrete casting. Thin films were made according to the research study of Mortensen [95]. Firstly, the dispersion of MWNT in aqueous solutions was made by using ultrasonication. Secondly, the Aquatec latex was added to produce a final MWNT-latex thin film that was further implemented, such as a spraying mixture by a Paasche airbrush.

During the fabrication process, Type I/II cement was used with a 0.42–0.44 w/c ratio together with SPL. The obtained fine aggregate from crushed granite was sieved by using a minimum size of 0.149 mm. A large coarse aggregate of river rock with a size of 19 mm was used. In order to achieve uniform coating, fine aggregates were sprayed six times, while coarse aggregates were sprayed three times together with manual mixing in order to prevent uncoated aggregate appearance. 

The cylinder and beam shaped specimens were cured for 28 days. Overall, four dif-ferent types of samples were made for the experiment, including control samples without coating, aggregate-coated, sand-coated and both sand and aggregated coated. The speci-mens were tested for compressive and flexural strengths. Detailed results are presented in Tables 1 and 2 of Reference [78].

According to test results, the lowest value for compressive strength was observed for the aggregate-coated sample and was found to be 6500 psi. Similarly, the values of sand-coated and both sand and aggregate-coated specimens were 6860 and 7580 psi. Hence, it can be said that the implementation of the MWNT-based thin films can save the mechanical properties of the untreated sample and even can improve it, as it can be observed from the specimen with both sand and aggregate-coated. The only disadvantage of coating is that the mechanical properties of coarse aggregates could be deteriorated due to the smoother surface formation that, in turn, would decrease the bonding effectiveness with the cement matrix. Nevertheless, it was found that the design guidelines were meet (4400 psi) by the samples.

From flexural testing, it was found that the aggregate-coated strength value (950 psi) was close to the control sample (953 psi). The difference in the modulus of rupture be-tween the control and the sand-coated sample was 6%, while a 14% difference was ob-tained from both sand and aggregate coated specimens.

Additionally, by using an electrical impedance tomography (EIT), three types of concrete plates (pristine concrete, aggregates coated plate with MWNT-latex thin films, sand-coated and both sand and aggregate-coated) were tested for damage detection. The first sample for testing was pristine concrete (control), where six copper mesh electrodes were located on each side of the plate. During the experiment, the damage was caused by three holes that were drilled inside of the concrete plate. According to the test results (Refer to Figure 7 in Reference [78]), the change in concrete resistivity was not pronounced. As the resistivity of the control concrete is high; therefore, the current that was applied did not spread throughout the entire concrete, thus resulting in poor damage detection.

A similar test procedure was performed for the second sample of aggregates coated plate with MWNT-latex thin films (Refer Figure 8 in Reference [78]). From the spatial resistivity map, it can be said that the damage detection failed in this sample as well due to an insufficient amount of MWNT after coating coarse aggregates that did not result in the creation of expected voltage response. 

The results for the last samples of sand-coated and both sand and aggregate-coated showed a clear image of EIT resistivity during the drilling process (Refer Figures 9 and 10 in Reference [78]). The authors noticed increase in resistivity at the same locations as the drilled holes. Moreover, the value of resistivity change was found to be larger for both sand and aggregate-coated concrete plate compared to only sand-coated with the MWNT-latex thin films. The reason for such an outcome could be the presence of a superior conductive interface; thus, the higher electrical conductivity appeared in both sand and aggregate-coated plate, unlike in the sand-coated plate.

**Figure 30:**
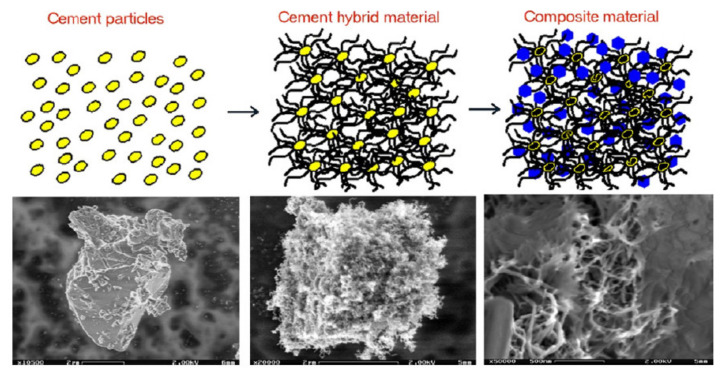
Schematic representation of CNT/CNF growing process. Reproduced with permission from [96], Elsevier, 2013.

Similarly, Nasibulin et al. [96] suggested a novel method to produce well-dispersed CNT or CNF by growing carbon nanomaterials directly on the surface of matrix particles without any catalyst. Figure 30 demonstrates the schematic process of growing carbon nanomaterials directly on top of the surface of matrix. This method resulted in the improvement of mechanical and electrical properties and also resolved issues regarding time consuming steps and shortening of CNT or CNF by ultrasonication. 

The first step is to perform a synthesis of CNT and CNF on the surface of cement and clinker particles, and the fluidized bed reactor was introduced for this purpose. After that, in order to find the concentration of grown CNT and CNF, samples were analyzed using thermogravimetric analysis. The final composite material, which is a hardened cement paste, can be formed after the hydration process. In order to investigate the compressive, flexural strengths and the electrical resistance, beams with dimensions of 60 mm × 10 mm × 10 mm were prepared and cured in aqueous condition for 7, 14 and 28 days. The researchers found that the utilization of cement particles as a catalyst results in compressive strength of the obtained cement paste value equal to more than two times the plain cement. Moreover, the electrical conductivity after water curing increased almost up to 40 times than the plain specimen. The research group also performed the testing of mechanical properties of mortar beams with dimensions of 40 mm × 40 mm × 160 mm that contained different concentrations of CNF in order to find the optimum amount of the CNF. Table 5 demonstrates the results obtained from the flexural and compressive strength tests for different samples after water curing for 28 days. The presented data revealed that the most optimum concentration of CNF is 0.4%, which also showed an increase in compressive strength of more than three times compared to the specimen without CNF. 

Overall, two new methods, including uniform dispersion of CNTs on top of the aggregate surface and the growth of carbon nanomaterials directly on the concrete matrix, were introduced. During the spraying of CNTs, the results showed that all of the specimens exceeded minimum design requirements for concrete; moreover, samples made of sand-coated and both sand and coarse aggregate-coated concrete showed higher compressive strength compared to the control specimen. The test for damage detection showed that the sand-coated and both sand and coarse aggregate-coated samples demonstrated localized resistivity results due to good propagation of current through the material. Similarly, the novel approach for synthesizing CNT/CNF on the surface of matrix particles demonstrated promising results in terms of compressive strength improvement as well as a high increase in electrical conductivity of the specimen. Therefore, these methods, i.e., spraying MWNT-latex thin films on top of the aggregates and growing CNT or CNF directly on the cement matrix, should be considered as one of the alternative methods for dispersion; however, more research studies should be conducted on different types of procedures adopted for nanomaterial distribution.

## 7. Applications of Self-Sensing Concrete

**Figure 31:**
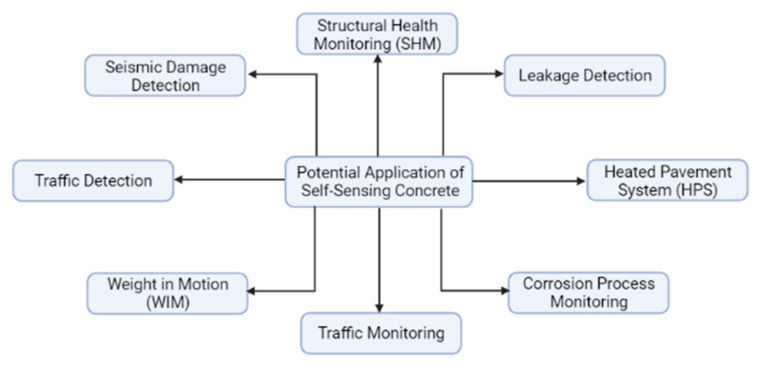
Potential fields of application.

Various researchers tried to implement SSC in real-life applications. Figure 31 demonstrates some of the areas where it has been applied. This section provides examples of the potential implementation of SSC in different fields.

**Figure 32:**
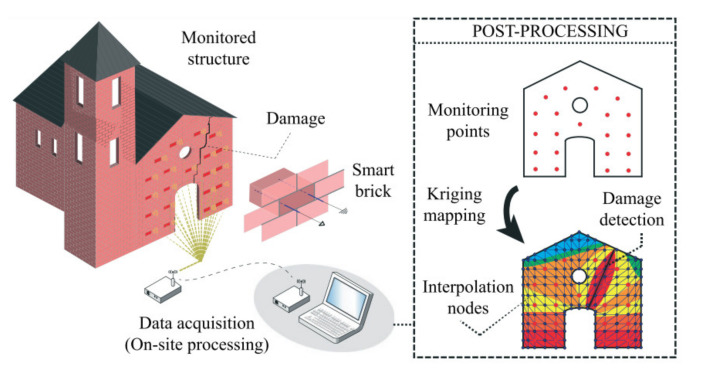
Structural health monitoring system using smart bricks. Reproduced with permission from [97], García-Macías E. et al., 2019.

There is a new method of monitoring masonry buildings by using the concept of SSC in the development of smart bricks. During the manufacturing process, special functional fillers are installed into the bricks that can be used as strain sensors, as shown in Figure 32 [97]. In this particular research study, a theoretical investigation of masonry shear wall and a 3D masonry building was conducted, during which it was found that smart bricks are effective for tracking strain variations. Different research studies that are based on simulations provided data that indicate the potential use of incorporated functional fillers into bricks for damage detection in new buildings, as well as in existing buildings [8,14]. Several researchers have also performed experiments in order to evaluate the performance and application of SSC. For instance, Alessandro et al. [8] performed experiments using single (one) brick, small-scale and medium-scale walls made of bricks to identify the potential performance of this technology in future practical applications. During the experimental procedure, the samples were subjected to eccentric compression. The results showed that the strain measurement capabilities of this technology are significantly improved when smart bricks are embedded in a brick wall, allowing even small changes in strain occurring inside the wall to be detected. Meoni et al. [14] also examined the performance of smart bricks inserted within masonry walls and found that smart bricks are sensitive to external stresses. Hence, smart brick technology has a very promising practical application. Alessandro et al. [98] investigated the potential use of this technology in seismic damage detection. The results from the eccentric compression load tests demonstrated that bricks can monitor changes in axial strain caused by increasing cracks, which was also confirmed by obtained data from numerical simulations. In addition to above mentioned characteristics, the cement-based composite is also resistant to factors such as changes in temperature and moisture, which allows smart bricks to be implemented as strain sensors, fire alarms and moisture sensors and, at the same time, serve as a load-bearing element of various structures [61]. Based on the above-mentioned research studies, it was concluded that smart bricks are a very promising novel sensing technology for structural health monitoring of masonry structures.

Another promising application of the concept of self-sensing technology is traffic detection and weight in motion. Shi and Chung [38] provided the laboratory demonstration for the traffic monitoring and weighing in motion using self-monitoring concrete with short carbon fibers. They identified that the resistance of the sample decreased reversibly with an increase in stress, and it was not dependent on the speed of the vehicle up to 55 mph. The data obtained suggest the effectiveness of this technology and confirmed its successful potential use. Chen and Chung [99] implemented the same method for traffic detection but by using carbon fibers inside the concrete. Similar conclusions were drawn by the researchers regarding the future potential of this technology as in the aforementioned source. Han et al. [11] investigated the characteristics of cement composite filled with piezoresistive MWNT fillers for traffic flow detection on the vehicle pavement. The results indicated that nanocomposite can be used for vehicular loadings and vehicle speed detection while in service for a weigh-in-motion measurement. In another research study, Han et al. [5] improved their traffic detection system by using pre-cast and cast-in-place concrete with self-sensing CNT sensors. To see the image of the road test, please refer to Figure 5 in Reference [5]. The authors identified that the novel method of CNT sensor implementation possess high detection, easy installation, long serviceability life, easy maintenance and good structural properties. The research conducted by Zhang et al. [100] evaluated the application of the self-sensing technique for weight monitoring during motion by embedding cement-based piezoelectric composites with the PMN ceramic in-pavement. For the experimental setup, please refer to Figure 4 in Reference [100]. The test results demonstrated precise sensing characteristics during vehicle passage with different loading parameters and speed at different temperature conditions both during the winter and summer. Another research study conducted by Han et al. [101] implemented sensors made of smart nickel particles as a vehicle detector. Embedded array of sensors inside the concrete pavement indicated that the proposed technology can accurately detect the passage of vehicles over the road and, hence, can be used in future projects. Graphite cement-based composite has also been used for traffic detection, monitoring and weight in motion. Birgin et al. [102] performed electromechanical tests on the samples containing graphite with a volume ratio of 12%, which was determined to be the most optimal content for cementitious materials. Additionally, the elastic properties of graphite cement-based composite could be related to traditional pavement materials. Composites with graphene inside concrete were also tested by Birgin et al. [103], and an alternative algorithm was developed during the experiment to determine the characteristics of weighing in motion, which provided evidence of the potential implementation of this technology. Overall, it can be said that the use of a graphite-cement composite material for practical use has great prospects due to its low cost and high durability. 

Potential usage of the hybrid glass/carbon textile-reinforced concrete (TRC) element was demonstrated by Goldfeld et al. [16]. According to the authors, the structural behavior of the element showed a clear response to the load, thus providing the possibility of using this sensor for structural health monitoring. Moreover, the sensory TRC element presented the sequential effect between wetting and cracking. Hence, capabilities demonstrated by the glass/carbon fiber-based textile reinforcement can be used to observe the functionality of different infrastructures, such as pipelines where the detection of leakage is essential. Gawel et al. [104] also performed an experiment in order to identify the feasibility of using cement with an embedded conductive filler for leak detection, as it is sensitive to mechanical stress. It was found that this material could be a good candidate for monitoring stress state changes and, therefore, has the potential for use in this area. Alkali-resistant (AR)-glass and carbon-based textile-reinforced concrete, which was implemented in the experiment conducted by Goldfeld and Perry [105], was also found as a preferred material for leakage detection application. It was identified that hybrid carbon-based textile-reinforced concrete can detect and discern the magnitude of wetting, as well as determine the severity of the surface cracking.

The implementation of self-sensing sensors could be used to monitor the corrosion process inside concrete elements. Lu [6] tracked the corrosion caused by corrosive solution ingress and by loading the RC beam. For this purpose, the authors put 3% of NaCl solution in plexiglass dams, which was placed at the top of the beam (center of the concrete beams). For the experimental setup, please refer to Figure 6 in Reference [6]. It was found that piezoelectric composite sensors have great potential to monitor the damage, including corrosion, due to good durability and high sensitivity. A similar experiment was performed by Fouad et al. [106] where they demonstrated that carbon fiber sensors installed on the concrete surface are suitable for locating and assessing corrosion of steel reinforcements with high accuracy. Overall, the laboratory experiment concluded that embedded cement-based piezoelectric sensors are appropriate for corrosion monitoring in concrete structures.

Removing the ice and snow from the pavement is traditionally performed by applying deicing salts and mechanical removal. However, the implementation of carbon fiber-based electrically conductive concrete (ECON) could help to create the heated pavement systems (HPS) as an alternative method to common methods. Sassani et al. [9] investigated and determined optimum carbon fiber dosage for HPS and analyzed this system by using finite element (FE) analysis. It was concluded that ECON could be highly effective in the application for melting ice or snow on top of the pavement. Similar conclusions were drawn in the research study performed by Sassani et al. [107], who also identified that the addition of a calcium nitrite-based corrosion inhibitor admixture as a conductivity-enhancing agent (CEA) enhanced electrical conductivity and compressive strength and was particularly effective for samples with low fiber content. In another research conducted by Sassani et al. [108], the authors investigated the feasibility of using polyurethane-carbon-microfiber (PU-CMF) composite coating for heating pavement in terms of durability, surface friction and volume conductivity. The authors found that the composite coating has great potential in the heat pavement system application; however, the only limitation was the construction cost, which can be reduced through detailed future studies.

One of the most common usages of SSC is for structural health monitoring. By comparing and analyzing the results from self-sensing abilities, it is possible to choose the best type as well as the optimal dosage of fillers. In one of the studies conducted by Feng and Ou [74], a self-sensing carbon fiber reinforced polymer (SCFRP) fabric that was embedded inside of the concrete was examined and analyzed for its self-sensing behavior under various applied loads. Experiments were carried out on several concrete beams with and without SCFRP. It was demonstrated that the yield strength of the specimen increased with the addition of SCFRP strengthening. Hence, the research study proposes to implement SCFRP fabric in the future due to its damage detection capability, as well as the strengthening ability. Another researcher, Sun et al. [15], investigated a cement-based strain sensor for ultra-high strength concrete (UHSC) health monitoring. The authors identified that an embedded cement-based strain sensor can sense stress and strain of the UHSC up to 154 MPa of stress. However, the main finding was that the cement-based strain sensor’s piezoresistive behavior includes three phases that include the following: high sensitive—linear phase; medium sensitive—nonlinear phase; low sensitive—a linear phase corresponding to an increase in load. Ubertini et al. [109] studied another material that can be used in SHM, which is the smart concrete doped with MWCNT. Experimental studies were carried out with the application of an impact load in the center of the beam and plate made of reinforced cement paste. The results clearly highlighted that the drop in the electrical response was detected at two points located across a crack allowing this material to be used for the localization as well as for direct damage detection. As in the research conducted by Downey et al. [36], cement composites doped with MWCNTs have promising applications for monitoring concrete structures [109].

Overall, there are a lot of fields that could use SSC technology in daily life. Table 6 provides summarized information on the potential application areas for SSC that were investigated by researchers. It can be observed that for seismic damage and crack detection, the researchers mainly used smart brick consisting of clay matrix and various conductive functional fillers. They performed experiments on a small-scale wall under loadings and/or using software tools for the simulation analysis [8,14,97,98]. By contrast, smart traffic monitoring was mainly performed using cement-based piezoelectric sensors that were embedded into the concrete pavement to measure the signals at the time when a vehicle passed over the sensor [5,11,38,100]. In the case of traffic detection, similar sensors as in smart traffic monitoring were implemented. Almost all researchers used pre-cast and cast-in-place SSC sensors made from different fillers that were installed inside the pavement to read the signal caused by vehicle movement [5,99,100,101]. A similar experimental pattern and material use was observed for weight in motion application [11,38,100,102,103]. In order to sense and detect leakage, the fillers dispersed inside the concrete and fiber-based textile reinforcement have been used [16,104,105]. Some researchers used embedded cement-based piezoelectric sensors for corrosion process monitoring [6]. However, less research studies have been performed in this area. Two different approaches were used for the realization of heated pavement systems (HPS): electrically conductive coating (ECOT) and electrically conductive Portland cement concrete made of self-sensing material [9,107,108]. Lastly, the researchers monitored structural health with a variety of methods, including filler dispersion, composite rods and textile sensors [15,22,29,41,74,75,85,109]. To summarize, the most popular sensors used for different fields of SSC are sensors made with dispersed fillers and embedded cement-based piezoelectric sensors.

## 8. Conclusions and Recommendations 

This paper provides an overview of SSC, which is an integration of electrically conductive filler material within cementitious materials. The self-sensing characteristics allow the composite to respond to stress/strain changes as well as to damage. Some of the benefits of using SSC include high sensitivity, the same lifespan as in concrete, good mechanical properties, easy installation and continuous monitoring.

Properties of SSC provide an ability to detect tiny cracks before they start to become significant, which is crucial in different civil infrastructure maintenance such as high-rise buildings, dams, pipelines, nuclear power plants, highways and bridges. However, there are various factors that affect the general characteristics and properties of SSC, such as functional filler concentration, moisture, geometric shape of functional filler, temperature and loading rate, etc.; hence, it is recommended to monitor and to control them in order to obtain accurate data and the desired concrete characteristics. It is also apparent that the researchers used different types of metrics, such as relative resistance, resistivity and conductivity to measure changes in the behavior of self-sensing concrete. While resistance can be easily measured with equipment, resistivity and conductivity require composite dimensions to obtain values. According to most studies, when reinforcement is presented in concrete, the electric current field is distorted, resulting in inaccuracy of resistivity measurements. Therefore, it is suggested that further research studies should include information that states considerations of relative to this factor.

In terms of functional fillers, there are many functional filler materials that were investigated and that could be incorporated into cement composites such as carbon nanotubes, carbon nanofibers, graphene oxide, carbon black, steel slag, nickel and graphite powder, magnetic fly ash and so on. The main aim of the research study regarding functional fillers was to find cheap and, at the same time, effective materials since it is the priciest component of SSC. Parameters such as dispersion method and mix preparation design should be considered in order to achieve the desired properties of concrete because they play a key role in improving electrical properties. Uniform dispersion of composites allows eliminating disadvantages such as a decrease in strength and poor sensing properties during agglomeration, providing the possibility to reduce the amount of filler and, thus, rendering the cost of SSC less expensive. Therefore, a reasonable selection of dispersion methods, including relatively new ones such as spraying, should be considered during the selection procedure in order to improve the uniformity of the mix. 

Although SSC has not been used in practical projects and its commercial applications are still very limited, there is a potential possibility to apply this technology in fields such as leakage detection, corrosion process monitoring, traffic monitoring and so on. Practical tests carried out on self-sensing materials, including carbon fibers, carbon nanomaterials and other carbon-based fillers, have been extensively described in detail and proven that this technology can be practically implemented in real-life projects. Despite differences in the obtained results from various experiments, the stress/strain change responses, as well as damage detection, are always clearly exhibited, demonstrating the potential of SSC in an application for structure health monitoring, weight in motion and traffic detection, etc. Overall, the most used materials that were optimal in different areas of application are sensors made with dispersed fillers and embedded cement-based piezoelectric sensors. However, in order to practically apply SSC in different structures and areas, additional research studies with a focus in creating universal standards in construction are suggested. 

## Figures and Tables

**Table 1:** Categories of functional fillers. Reproduced with permission from [17], Abdullah et al., 2019.

Classification			Typical Functional Filler
Criteria	Categories		
Material component	Carbonaceous		CF, CNF, CNT, CB, GP, nanoscale GP
	Metal or metal oxide		SF, NP, nano TiO_2_, nano Fe_2_O_3_
Filler shape	Fibrous		CF, SF, CNF, CNT
	Particle		CB, GP, NP
Filler scale	Macroscale		SF, SS
	Microscale		CF, GP, NP
	Nanoscale		CNF, CNT, nano TiO_2_, nano Fe_2_O_3_
Conductive capability	Electrically conductive		CF, SF, CNF, CNT, CB, NP
	Semi-conductive		Nano TiO_2_, nano Fe_2_O_3_, SS, MFA
	Nonconducting		Short PVAF
Application stile	Single		CF, CNT, CB, SS, NP
	Hybrid	Fibers	CF & CNT, Copper-coated CF & SF, PVAF & SF, PVAF & CF
		Fibers and particles	CF & CB, CF & GP, SF & GP, CNT & CB, Iron containing conductive functional aggregate and CF, PVAF & CB
		Particles	CB & NP, MFA & SS
Surface state	Regular		CF, CNT
	Modified		Ozone-treated CF, CF treated with concentrated nitric acid, carbon-coated nylon fiber, copper-coated CF, sodium hypochlorite-treated CF, CNT treated with a mixed solution of H_2_SO_4_ and HNO_3_

Note: Short and Continuous Carbon Fibers (CF), Carbon Nanofibers (CNF), Carbon Nanotubes (CNT), Carbon Black (CB), Graphite powder (GP), Steel Fiber (SF), Nickel Powder (NP), Steel Slag (SS), Magnetic Fly Ash (MFA), Short Polyvinyl Alcohol Fiber (PVAF).

**Table 2:** Chemical dispersion materials. Reproduced with permission from [4], Elsevier, 2015.

Category	Dispersion Material	Dispersion Functional Fillers
Surfactant	Water-reducing agent Methylcellulose (MC) Carboxy MC Hydroxypropyl MCSodium dodecyl sulfate (SDS) Sodium dodecylbenzene sulfonate (SDBS) SDBS and polyacrylic acid	CF, CNT, CNF, CB, GP NP, Nano Fe_2_O_3_ CF, CNT, CF CNT, CNF CNT
Minerals	Admixture CF, CB, SF, NP	Silica fume

**Table 3:** Optimal Filler Concentration.

Matrix	Filler	Optimal Concentration	Reference
Paste	CF	0.63% by mass	[27]
Mortar	CF	0.5% by volume	[28]
Concrete	CNT	6 wt% of cement	[29]
Paste	CNT	0.1 wt%	[30]
Mortar	CNT	0.3 wt%	[31]
Concrete	CNF	1% by volume	[32]
Paste	CNF	1.25 vol%	[33]

**Table 4:** Overview of different functional fillers covered in the literature.

	Matrix	Fillers	Optimal Filler Concentration	Dispersion Area	Reference
Carbon Short Fiber-Based Self-Sensing Concrete	Paste	Carbon Short Fiber	0.5 vol% of cement	Paste	[40]
	Concrete	Carbon Short Fiber	-	Bottom Layer	[72]
Carbon Continuous Fiber-Based Self-Sensing Concrete	Concrete	Hybrid: 1st filler-Carbon Fiber 2nd filler-Glass Fiber	1st filler—0.2% 2nd filler—48% (*)	Rod	[73]
	Concrete	Carbon Fiber Reinforced Polymer	-	Reinforcing Material	[74]
	Concrete	Continuous Carbon Fiber	Total fiber fraction—0.42 vol (*)	Reinforcing Material	[75]
Carbon Nanomaterials for Self-Sensing Concrete	Paste	Sample: Carbon Nanotube Carbon Nanofiber Carbon Black Graphene Nanoplatelets	1 mass% of cement	Paste	[76]
	Paste	Carbon Nanofibers	2 mass% of cement	Paste	[10]
	Concrete	Hybrid: Internal filler-Carbon Nanoparticles External filler-Glass Fibers Reinforced Polymers	Internal filler—5 wt% (*)	Rod	[77]
	Concrete	Hybrid: 1st filler-Carbon Nanotube 2nd filler-Nano Carbon Black	6 wt% of cement	Concrete	[29]
	Paste	Hybrid: 1st filler-Carbon Nanotube 2nd filler-Carbon Fiber	1st filler—1 vol% 2nd filler—15 vol% (*)	Paste	[26]
	Concrete	Carbon Nanotube	2 wt% (*)	Thin Film on Top of the Aggregates	[78]

* Complete details are not available.

**Table 5:** Mechanical properties of samples of CNF/clinker composite. Reproduced with permission from [96], Elsevier, 2013.

Test Number	CNF Concentration, %	Flexural Strength, Mpa	Compressive Strength, Mpa
1	0	5.8	58
1	0.4	6	172
1	1.5	5.1	58
1	5	1.6	20
2	0	6.7	87
2	0.2	7.2	130
2	0.4	7.1	217
2	0.5	7.4	138

**Table 6:** Potential applications of self-sensing concrete.

Implementation Filed	Sensor	Characteristics	Material	Literature
Seismic damage and crack detection	Smart bricks	New as well as existing buildings with smart piezoresistive bricks have potentially effective and durable technology that can be used to provide critical information for structural prognosis and long-term structural health monitoring.	Clay martix plus TiO_2_	[8,97]
			Clay martix plus MWCNTs, Clay martix plus CNFs, Clay martix plus GNPs	[14]
			Clay martix plus to steel micro-fibers	[98]
Smart traffic monitoring	Embedded cement-based piezoelectric sensors	Sensors demonstrated good relationships between compressive stress and electrical resistance together with a high level of piezoelectric sensitivity.	MWCNTs filled cement composite	[11]
			Composite with the PMN ceramic and concrete	[100]
		Self-sensing concrete technology has great potential in detecting traffic data. This would be beneficial for traffic monitoring, management and weighing in motion.	Concrete plus CNT	[5]
			Concrete plus short CF	[38]
Traffic detection	Pre-cast and cast-in-place self-sensing concrete sensors	The sensor demonstrated remarkable sensing capabilities and low false-alarm rate during variations in the speed of a vehicle and outside temperature. Smart concrete has good potential in non-destructive flow detection.	Concrete plus CNT	[5]
			Nickel particle filled cement-based sensors embedded into a concrete	[101]
			Composite with the PMN ceramic and concrete	[100]
			Concrete plus CF	[99]
Weight in motion (WIM)	Embedded cement-based piezoelectric sensors	The weight of the car could be determined by considering its speed. Sensors have high piezoelectric sensitivity, high durability and linear mechanical-electrical response.	Composite with graphite and concrete	[102,103]
			MWCNTs filled cement composite	[11]
			Concrete plus short CF	[38]
			Composite with the PMN ceramic and concrete	[100]
Leakage detection	Dispersed fillers	Cement with an embedded conductive filler demonstrated great potential in sensing changes in the mechanical environment such as for leakage detection.	Cement plus CNF	[104]
	Fiber-based textile reinforcement	Hybrid demonstrated good structural response in pre-cracked linear and post-cracking behavior. Additionally, all wetting events of the cracked beam were captured using this sensory textile, thus showing its usage for leakage detection.	Alkali-resistant (AR)-glass and carbon-based textile-reinforced concrete	[105]
			Glass/carbon fiber–based textile reinforcement	[16]
Corrosion process monitoring	Embedded cement-based piezoelectric sensors	Piezoelectric composite sensors are very suitable for monitoring the corrosion process in concrete structures.	Embedded cement-based piezoelectric sensor	[6]
Heated pavement systems (HPS)	Electrically Conductive Coating (ECOT)	CMF dosage in the range of 10 to 15% is the most desirable. This technology of PU-CMF implementation is a potential topic for future studies.	Polyurethane-carbon-microfiber (PU-CMF) composite coating	[108]
	Electrically Conductive Portland Cement Concrete	ECON demonstrated its effective application during ice/snow melting. Optimum carbon fiber dosage in concrete was found to be 0.75% (Vol.)	CF based electrically conductive concrete (ECON)	[9,107]
Structural health monitoring	Dispersed fillers	The piezoresistive behavior of cement-based sensors demonstrated three different phases with the increase in loading.	Cement-based strain sensor (CBSS)	[15]
		There is a great impact of using smart concrete in SHM which demonstrated the revolutionary potential in the structural strengthening together with the self-sensing capacity.	Concrete plus MWCNT	[41,109]
			Carbon Fiber Reinforced Polymer (CFRP)	[74]
			Concrete plus CNT	[22]
			Concrete plus CNT/NCB	[29]
	Composite rods	Piezoresistive behaviour of BCRs can be useful in the future to determine the damage that was caused to the structure.	Core-reinforced braided composite rods (BCRs)	[85]
	Textile sensors	Strengthening fabrics can be applied for strengthening and monitoring different types of structures and can also be used as self-sensors for SHM. The advantages of this technology are low cost, light weight and versatility of the material.	Carbon Fiber Epoxy-Matrix Composite	[75]

## References

[1] Konkanov M., Salem T., Jiao P., Niyazbekova R., Lajnef N. (2020). Environment-Friendly, Self-Sensing Concrete Blended with Byproduct Wastes. Sensors.

[2] Dong W., Li W., Tao Z., Wang K. (2019). Piezoresistive properties of cement-based sensors: Review and perspective. Constr. Build. Mater..

[3] Ou J., Han B. (2009). Piezoresistive Cement-based Strain Sensors and Self-sensing Concrete Components. J. Intell. Mater. Syst. Struct..

[4] Han B., Ding S., Yu X. (2015). Intrinsic self-sensing concrete and structures: A review. Measurement.

[5] Han B., Zhang K., Burnham T., Kwon E., Yu X. (2013). Integration and road tests of a self-sensing CNT concrete pavement system for traffic detection. Smart Mater. Struct..

[6] Lu Y., Zhang J., Li Z., Dong B. (2013). Corrosion monitoring of reinforced concrete beam using embedded cement-based piezoelectric sensor. Mag. Concr. Res..

[7] Wen S., Chung D. (2001). Carbon fiber-reinforced cement as a strain-sensing coating. Cem. Concr. Res..

[8] Downey A., D’Alessandro A., Laflamme S., Ubertini F. (2017). Smart bricks for strain sensing and crack detection in masonry structures. Smart Mater. Struct..

[9] Sassani A., Arabzadeh A., Ceylan H., Kim S., Sadati S.S., Gopalakrishnan K., Taylor P.C., Abdualla H. (2018). Carbon fiber-based electrically conductive concrete for salt-free deicing of pavements. J. Clean. Prod..

[10] Galao O., Baeza F.J., Zornoza E., Garcés P. (2013). Strain and damage sensing properties on multifunctional cement composites with CNF admixture. Cem. Concr. Compos..

[11] Han B., Yu X., Kwon E. (2009). A self-sensing carbon nanotube/cement composite for traffic monitoring. Nanotechnology.

[12] Spinelli G., Lamberti P., Tucci V., Guadagno L., Vertuccio L. (2020). Damage Monitoring of Structural Resins Loaded with Carbon Fillers: Experimental and Theoretical Study. Nanomaterials.

[13] Murray C.M., Doshi S.M., Sung D.H., Thostenson E.T. (2020). Hierarchical Composites with Electrophoretically Deposited Carbon Nanotubes for In Situ Sensing of Deformation and Damage. Nanomaterials.

[14] D’Alessandro A., Ubertini F., Downey A., Laflamme S., Meoni A., Sohn H. (2018). Strain monitoring in masonry structures using smart bricks. Proceedings of the Sensors and Smart Structures Technologies for Civil, Mechanical, and Aerospace Systems 2018.

[15] Sun M.-Q., Liew R., Zhang M.-H., Li W. (2014). Development of cement-based strain sensor for health monitoring of ultra high strength concrete. Constr. Build. Mater..

[16] Goldfeld Y., Rabinovitch O., Fishbain B., Quadflieg T., Gries T. (2015). Sensory carbon fiber based textile-reinforced concrete for smart structures. J. Intell. Mater. Syst. Struct..

[17] Abdullah W., Mohammed A., Abdullah A. (2019). Self-Sensing Concrete: A Brief Review. Int. J. Adv. Mech. Civil Eng..

[18] Faghih F. (2018). Structural Performance of Nano Concrete-Steel.

[19] Wen S., Chung D. (2007). Electrical-resistance-based damage self-sensing in carbon fiber reinforced cement. Carbon.

[20] Heilig M.L. (1994). United States Patent Office. ACM SIGGRAPH Comput. Graph..

[21] Uri D., Ackermann K.C. (2018). Self-Sensing Concrete for Structural Health Monitoring of Smart Infrastructures. Master’s Thesis.

[22] D’Alessandro A., Rallini M., Ubertini F., Materazzi A.L., Kenny J.M. (2016). Investigations on scalable fabrication procedures for self-sensing carbon nanotube cement-matrix composites for SHM applications. Cem. Concr. Compos..

[23] Wang L., Loh K.J., Brely L., Bosia F., Pugno N.M. (2016). An experimental and numerical study on the mechanical properties of carbon nanotube-latex thin films. J. Eur. Ceram. Soc..

[24] Han B., Zhang K., Yu X., Kwon E., Ou J. (2012). Fabrication of Piezoresistive CNT/CNF Cementitious Composites with Superplasticizer as Dispersant. J. Mater. Civ. Eng..

[25] Li H., Xiao H.-G., Ou J.-P. (2004). A study on mechanical and pressure-sensitive properties of cement mortar with nanophase materials. Cem. Concr. Res..

[26] Azhari F., Banthia N. (2012). Cement-based sensors with carbon fibers and carbon nanotubes for piezoresistive sensing. Cem. Concr. Compos..

[27] Baeza F.J., Galao O., Zornoza E., Garces P. (2013). Effect of aspect ratio on strain sensing capacity of carbon fiber reinforced cement composites. Mater. Des..

[28] Nguyen D.-L., Kim D.-J., Thai D.-K. (2019). Enhancing Damage-Sensing Capacity of Strain-Hardening Macro-Steel Fiber-Reinforced Concrete by Adding Low Amount of Discrete Carbons. Materials.

[29] Ding S., Wang Y.-W., Ni Y.-Q., Han B. (2020). Structural modal identification and health monitoring of building structures using self-sensing cementitious composites. Smart Mater. Struct..

[30] Han B., Yu X., Kwon E., Ou J. (2011). Effects of CNT concentration level and water/cement ratio on the piezoresistivity of CNT/cement composites. J. Compos. Mater..

[31] Liu C., Liu G., Ge Z., Guan Y., Cui Z., Zhou J. (2019). Mechanical and Self-Sensing Properties of Multiwalled Carbon Nanotube-Reinforced ECCs. Adv. Mater. Sci. Eng..

[32] Howser R., Dhonde H.B., Mo Y. (2011). Self-sensing of carbon nanofiber concrete columns subjected to reversed cyclic loading. Smart Mater. Struct..

[33] Wang H., Gao X., Wang R. (2017). The influence of rheological parameters of cement paste on the dispersion of carbon nanofibers and self-sensing performance. Constr. Build. Mater..

[34] Tian Z., Li Y., Zheng J., Wang S. (2019). A state-of-the-art on self-sensing concrete: Materials, fabrication and properties. Compos. Part. B: Eng..

[35] Mo Y., Howser R., Maguire R. (2013). Carbon Nanofiber Concrete for Damage Detection of Infrastructure.

[36] Downey A., Garcia-Macias E., D’Alessandro A., Laflamme S., Castro-Triguero R., Ubertini F., Wu H.F., Gyekenyesi A.L., Shull P.J., Yu T.-Y. (2017). Continuous and embedded solutions for SHM of concrete structures using changing electrical potential in self-sensing cement-based composites. Proceedings of the Nondestructive Characterization and Monitoring of Advanced Materials, Aerospace, and Civil Infrastructure 2017.

[37] Wen S., Chung D.D.L. (2007). Piezoresistivity-Based Strain Sensing in Carbon Fiber-Reinforced Cement. ACI Mater. J..

[38] Shi Z.-Q., Chung D. (1999). Carbon fiber-reinforced concrete for traffic monitoring and weighing in motion. Cem. Concr. Res..

[39] Chiarello M., Zinno R. (2005). Electrical conductivity of self-monitoring CFRC. Cem. Concr. Compos..

[40] Wen S., Chung D. (2006). Self-sensing of flexural damage and strain in carbon fiber reinforced cement and effect of embedded steel reinforcing bars. Carbon.

[41] Materazzi A., Ubertini F., D’Alessandro A. (2013). Carbon nanotube cement-based transducers for dynamic sensing of strain. Cem. Concr. Compos..

[42] Li H., Xiao H.-G., Ou J.-P. (2006). Effect of compressive strain on electrical resistivity of carbon black-filled cement-based composites. Cem. Concr. Compos..

[43] Xie P., Gu P., Beaudoin J.J. (1996). Electrical percolation phenomena in cement composites containing conductive fibres. J. Mater. Sci..

[44] Han B., Yu X., Ou J. (2014). Sensing Mechanisms of Self-Sensing Concrete. Self-Sensing Concrete in Smart Structures.

[45] Li G.Y., Wang P.M., Zhao X. (2007). Pressure-sensitive properties and microstructure of carbon nanotube reinforced cement composites. Cem. Concr. Compos..

[46] Chung D.D.L. (2002). Piezoresistive Cement-Based Materials for Strain Sensing. J. Intell. Mater. Syst. Struct..

[47] Han B., Wang Y., Sun S., Yu X., Ou J. (2014). Nanotip-induced ultrahigh pressure-sensitive composites: Principles, properties and applications. Compos. Part. A Appl. Sci. Manuf..

[48] Han B., Zhang K., Yu X., Kwon E., Ou J. (2012). Electrical characteristics and pressure-sensitive response measurements of carboxyl MWNT/cement composites. Cem. Concr. Compos..

[49] Saafi M. (2009). Wireless and embedded carbon nanotube networks for damage detection in concrete structures. Nanotechnology.

[50] Mishnaevsky L., Dai G. (2013). Hybrid carbon/glass fiber composites: Micromechanical analysis of structure–damage resistance relationships. Comput. Mater. Sci..

[51] Ding S., Dong S., Ashour A., Han B. (2019). Development of sensing concrete: Principles, properties and its applications. J. Appl. Phys..

[52] Han B., Yu X., Ou J. (2014). Sensing Properties of Self-Sensing Concrete. Self-Sensing Concrete in Smart Structures.

[53] Meehan D.G., Wang S., Chung D. (2009). Electrical-resistance-based Sensing of Impact Damage in Carbon Fiber Reinforced Cement-based Materials. J. Intell. Mater. Syst. Struct..

[54] Reza F., A Yamamuro J., Batson G.B. (2004). Electrical resistance change in compact tension specimens of carbon fiber cement composites. Cem. Concr. Compos..

[55] Wang X., Wang Y., Jin Z. (2002). Electrical conductivity characterization and variation of carbon fiber reinforced cement composite. J. Mater. Sci..

[56] Chen B., Liu J. (2008). Damage in carbon fiber-reinforced concrete, monitored by both electrical resistance measurement and acoustic emission analysis. Constr. Build. Mater..

[57] Mc Carter W.J., Starrs G., Chrisp T.M., Banfill P.F.G. (2007). Activation energy and conduction in carbon fibre reinforced cement matrices. J. Mater. Sci..

[58] Lin V.W.J., Li M., Lynch J.P., Li V.C. (2011). Mechanical and Electrical Characterization of Self-Sensing Carbon Black ECC. Nondestructive Characterization for Composite Materials, Aerospace Engineering, Civil. Infrastructure, and Homeland Security 2011.

[59] Chen B., Liu J., Wu K. (2005). Electrical responses of carbon fiber reinforced cementitious composites to monotonic and cyclic loading. Cem. Concr. Res..

[60] Li H., Ou J.P. (2008). Smart Concrete, Sensors and Self-Sensing Concrete Structures. Key Eng. Mater..

[61] Demircilioğlu E., Teomete E., Schlangen E., Baeza F.J. (2019). Temperature and moisture effects on electrical resistance and strain sensitivity of smart concrete. Constr. Build. Mater..

[62] Chacko R.M., Banthia N., A Mufti A. (2007). Carbon-fiber-reinforced cement-based sensors. Can. J. Civ. Eng..

[63] Cao J., Chung D. (2002). Effect of strain rate on cement mortar under compression, studied by electrical resistivity measurement. Cem. Concr. Res..

[64] Hanxun B., Yu X., Zhang K., Kwon E., Ou J. (2011). Sensing properties of CNT-filled cement-based stress sensors. J. Civ. Struct. Health Monit..

[65] Maier M. (2020). The effect of moisture and reinforcement on the self-sensing properties of hybrid-fiber-reinforced concrete. Eng. Res. Express.

[66] Wen S., Chung D. (2006). The role of electronic and ionic conduction in the electrical conductivity of carbon fiber reinforced cement. Carbon.

[67] Li H., Xiao H., Ou J. (2008). Electrical property of cement-based composites filled with carbon black under long-term wet and loading condition. Compos. Sci. Technol..

[68] Cai H., Liu X. (1998). Freeze-thaw durability of concrete: Ice formation process in pores. Cem. Concr. Res..

[69] Azhari F. (2008). Cement-Based Sensors for Structural Health Monitoring. Master’s Theses.

[70] Cao J., Chung D. (2002). Damage evolution during freeze–thaw cycling of cement mortar, studied by electrical resistivity measurement. Cem. Concr. Res..

[71] Demirel B., Yazicioğlu S., Orhan N. (2006). Electrical behaviour of carbon fibre-reinforced concrete with increasing loading in varying and constant frequencies. Mag. Concr. Res..

[72] Wang W., Dai H., Wu S. (2008). Mechanical behavior and electrical property of CFRC-strengthened RC beams under fatigue and monotonic loading. Mater. Sci. Eng. A.

[73] Nanni F., Ruscito G., Forte G., Gusmano G. (2007). Design, manufacture and testing of self-sensing carbon fibre–glass fibre reinforced polymer rods. Smart Mater. Struct..

[74] Feng Q., Ou J. (2018). Self-Sensing CFRP Fabric for Structural Strengthening and Damage Detection of Reinforced Concrete Structures. Sensors.

[75] Salvado R., Lopes C., Szojda L., Araujo P., Gorski M., Velez F.J., Castro-Gomes J., Krzywon R. (2015). Carbon Fiber Epoxy Composites for Both Strengthening and Health Monitoring of Structures. Sensors.

[76] D’Alessandro A., Meoni A., Ubertini F. (2018). Innovative Composites with Carbon Nanofillers for Self-Sensing Structural RC Beams. Nano Hybrids Compos..

[77] Nanni F., Ruscito G., Nad L., Gusmano G. (2009). Self-sensing Nanocomposite CnP—GFRP Rods as Reinforcement and Sensors of Concrete Beams. J. Intell. Mater. Syst. Struct..

[78] Gupta S., Gonzalez J.G., Loh K.J. (2016). Self-sensing concrete enabled by nano-engineered cement-aggregate interfaces. Struct. Health Monit..

[79] Huang X. (2009). Fabrication and Properties of Carbon Fibers. Materials.

[80] Xu Z., Gao C. (2015). Graphene fiber: A new trend in carbon fibers. Mater. Today.

[81] Rana S., P S., Fangueiro R., Correia A.G. (2016). A review on smart self-sensing composite materials for civil engineering applications. AIMS Mater. Sci..

[82] Park S.-J., Seo M.-K., Thomas S., Kuruvilla J., Malhotra S.K., Goda K., Sreekala M.S. (2012). Carbon Fiber-Reinforced Polymer Composites: Preparation, Properties, and Applications. Proceedings of the Polymer Composites 2012.

[83] Markovičová L., Zatkalíková V., Hanusová P. (2019). Carbon Fiber Polymer Composites. Qual. Prod. Improv.—QPI.

[84] Swolfs Y., Meerten Y., Hine P., Ward I., Verpoest I., Gorbatikh L. (2015). Introducing ductility in hybrid carbon fibre/self-reinforced composites through control of the damage mechanisms. Compos. Struct..

[85] Rana S., Zdraveva E., Pereira C.G., Fangueiro R., Correia A.G. (2014). Development of Hybrid Braided Composite Rods for Reinforcement and Health Monitoring of Structures. Sci. World J..

[86] Gonilho-Pereira C., Jalali S., Fangueiro R., Araújo M., Marques P. (2010). Hybrid composite rods for concrete reinforcement. Structures & Architecture.

[87] Inoue R., Arai Y., Kubota Y., Goto K., Kogo Y. (2018). Development of short- and continuous carbon fiber-reinforced ZrB2-SiC-ZrC matrix composites for thermal protection systems. Ceram. Int..

[88] Saidi M., Gabor A. (2020). Experimental analysis and analytical modelling of the textile/matrix interface shear stress in textile reinforced cementitious matrix composites. Compos. Part. A Appl. Sci. Manuf..

[89] D’Alessandro A., Ubertini F., Laflamme S., Materazzi A.L. (2016). Towards smart concrete for smart cities: Recent results and future application of strain-sensing nanocomposites. J. Smart Cities.

[90] Galao O., Baeza F.J., Zornoza E., Garcés P. (2017). Carbon Nanofiber Cement Sensors to Detect Strain and Damage of Concrete Specimens Under Compression. Nanomaterials.

[91] Han B., Zhang L., Sun S., Yu X., Dong X., Wu T., Ou J. (2015). Electrostatic self-assembled carbon nanotube/nano carbon black composite fillers reinforced cement-based materials with multifunctionality. Compos. Part. A Appl. Sci. Manuf..

[92] Zhang L., Ding S., Li L., Dong S., Wang D., Yu X., Han B. (2018). Effect of characteristics of assembly unit of CNT/NCB composite fillers on properties of smart cement-based materials. Compos. Part. A Appl. Sci. Manuf..

[93] Kim K.J., Yu W.-R., Lee J.S., Gao L., Thostenson E.T., Chou T.-W., Byun J.-H. (2010). Damage characterization of 3D braided composites using carbon nanotube-based in situ sensing. Compos. Part. A Appl. Sci. Manuf..

[94] Wan L., Guo J. (2015). Damage Analysis of Three-Dimensional Braided Composite Material Using Carbon Nanotube Threads. Exp. Tech..

[95] Mortensen L.P., Ryu D.H., Zhao Y.J., Loh K.J. (2013). Rapid Assembly of Multifunctional Thin Film Sensors for Wind Turbine Blade Monitoring. Key Eng. Mater..

[96] Nasibulin A.G., Koltsova T., Nasibulina L.I., Anoshkin I.V., Semencha A., Tolochko O.V., Kauppinen E. (2013). A novel approach to composite preparation by direct synthesis of carbon nanomaterial on matrix or filler particles. Acta Mater..

[97] García-Macías E., Ubertini F. (2018). Earthquake-induced damage detection and localization in masonry structures using smart bricks and Kriging strain reconstruction: A numerical study. Earthq. Eng. Struct. Dyn..

[98] Alessandro A.D., Meoni A., Ubertini F. Recent results on the use of smart bricks for earthquake-induced damage detection in masonry structures. Proceedings of the XVIII ANIDIS Conference, Seismic Engineering in Italy, Pisa University Press.

[99] Chen P.-W., Chung D.D.L. (1993). Carbon fiber reinforced concrete for smart structures capable of non-destructive flaw detection. Smart Mater. Struct..

[100] Zhang J., Lu Y., Lu Z., Liu C., Sun G., Li Z. (2015). A new smart traffic monitoring method using embedded cement-based piezoelectric sensors. Smart Mater. Struct..

[101] Han B., Zhang K., Yu X., Kwon E., Ou J. (2011). Nickel particle-based self-sensing pavement for vehicle detection. Measurement.

[102] Birgin H.B., D’Alessandro A., Laflamme S., Ubertini F. (2020). Smart Graphite–Cement Composite for Roadway-Integrated Weigh-In-Motion Sensing. Sensors.

[103] Birgin H.B., Laflamme S., D’Alessandro A., Garcia-Macias E., Ubertini F. (2020). A Weigh-in-Motion Characterization Algorithm for Smart Pavements Based on Conductive Cementitious Materials. Sensors.

[104] Gawel K., Szewczyk D., Cerasi P. (2021). Self-Sensing Well Cement. Materials.

[105] Goldfeld Y., Perry G. (2018). AR-glass/carbon-based textile-reinforced concrete elements for detecting water infiltration within cracked zones. Struct. Health Monit..

[106] Fouad N., A Saifeldeen M., Huang H., Wu Z. (2017). Corrosion monitoring of flexural reinforced concrete members under service loads using distributed long-gauge carbon fiber sensors. Struct. Health Monit..

[107] Sassani A., Ceylan H., Kim S., Gopalakrishnan K., Arabzadeh A., Taylor P.C. (2017). Influence of mix design variables on engineering properties of carbon fiber-modified electrically conductive concrete. Constr. Build. Mater..

[108] Sassani A., Arabzadeh A., Ceylan H., Kim S., Gopalakrishnan K., Taylor P.C., Nahvi A. (2019). Polyurethane-carbon microfiber composite coating for electrical heating of concrete pavement surfaces. Heliyon.

[109] Ubertini F., D’Alessandro A., Downey A., García-Macías E., Laflamme S., Castro-Triguero R. (2017). Recent Advances on SHM of Reinforced Concrete and Masonry Structures Enabled by Self-Sensing Structural Materials. Multidiscip. Digit. Publ. Inst. Proc..

